# A robust approach to estimate relative phytoplankton cell abundances from metagenomes

**DOI:** 10.1111/1755-0998.13592

**Published:** 2022-02-16

**Authors:** Juan José Pierella Karlusich, Eric Pelletier, Lucie Zinger, Fabien Lombard, Adriana Zingone, Sébastien Colin, Josep M. Gasol, Richard G. Dorrell, Nicolas Henry, Eleonora Scalco, Silvia G. Acinas, Patrick Wincker, Colomban de Vargas, Chris Bowler

**Affiliations:** ^1^ Institut de Biologie de l'ENS (IBENS), Département de Biologie, École normale supérieure, CNRS, INSERM Université PSL Paris France; ^2^ CNRS Research Federation for the study of Global Ocean Systems Ecology and Evolution FR2022/Tara Oceans GOSEE Paris France; ^3^ Génomique Métabolique, Genoscope, Institut François Jacob, CEA, CNRS, Univ Evry Université Paris‐Saclay Evry France; ^4^ CNRS, Laboratoire d'Océanographie de Villefranche (LOV) Sorbonne Universités Villefranche‐sur‐Mer France; ^5^ Institut Universitaire de France (IUF) Paris France; ^6^ Stazione Zoologica Anton Dohrn Villa Comunale Naples Italy; ^7^ European Molecular Biology Laboratory Heidelberg Germany; ^8^ CNRS, Station Biologique de Roscoff, UMR 7144, ECOMAP Sorbonne Université Roscoff France; ^9^ Max Planck Institute for Developmental Biology Tübingen Germany; ^10^ Department of Marine Biology and Oceanography Institut de Ciènces del Mar CSIC Barcelona Spain; ^11^ CNRS, FR2424, ABiMS, Station Biologique de Roscoff Sorbonne Université Roscoff France

**Keywords:** 18S rRNA, metabarcoding, metagenomics, metatranscriptomics, photosynthesis, phytoplankton, *psbO*, *Tara* Oceans

## Abstract

Phytoplankton account for >45% of global primary production, and have an enormous impact on aquatic food webs and on the entire Earth System. Their members are found among prokaryotes (cyanobacteria) and multiple eukaryotic lineages containing chloroplasts. Genetic surveys of phytoplankton communities generally consist of PCR amplification of bacterial (16S), nuclear (18S) and/or chloroplastic (16S) rRNA marker genes from DNA extracted from environmental samples. However, our appreciation of phytoplankton abundance or biomass is limited by PCR‐amplification biases, rRNA gene copy number variations across taxa, and the fact that rRNA genes do not provide insights into metabolic traits such as photosynthesis. Here, we targeted the photosynthetic gene *psbO* from metagenomes to circumvent these limitations: the method is PCR‐free, and the gene is universally and exclusively present in photosynthetic prokaryotes and eukaryotes, mainly in one copy per genome. We applied and validated this new strategy with the size‐fractionated marine samples collected by *Tara* Oceans, and showed improved correlations with flow cytometry and microscopy than when based on rRNA genes. Furthermore, we revealed unexpected features of the ecology of these ecosystems, such as the high abundance of picocyanobacterial aggregates and symbionts in the ocean, and the decrease in relative abundance of phototrophs towards the larger size classes of marine dinoflagellates. To facilitate the incorporation of *psbO* in molecular‐based surveys, we compiled a curated database of >18,000 unique sequences. Overall, *psbO* appears to be a promising new gene marker for molecular‐based evaluations of entire phytoplankton communities.

## Abstract 2

see also the Perspective by Georgina L. Brennan

## 1 INTRODUCTION

Photosynthetic plankton, or phytoplankton, consist of unicellular organisms of diverse evolutionary history and ecology. They are responsible for more than 45% of Earth's primary production (Field et al., 1998), fuelling aquatic food webs, microbial decomposition, and the global ocean biological carbon pump (Guidi et al., 2009). They include prokaryotes (cyanobacteria) and multiple eukaryotic lineages that acquired photosynthesis either through the primary endosymbiosis of cyanobacteria, or (and predominantly) through secondary and higher endosymbioses of eukaryotic algae (Pierella Karlusich et al., 2020). They display a broad body size spectrum, from less than 1 micron (e.g., *Prochlorococcus*, *Ostreococcus*) to several millimetres (e.g., *Trichodesmium* colonies, colonial green algae, and chain‐forming diatoms), either due to cell size variation, aggregation or symbioses (Beardall et al., 2009). This size variability partly explains their different roles in the food web and in the biological carbon pump. For example, cyanobacteria are generally thought to be recycled within the microbial loop, whereas larger eukaryotic phytoplankton are usually considered more important in energy transfer to higher trophic levels (through grazing by small protists, zooplankton, and/or larvae [Ullah et al., 2018]) and in sequestering atmospheric CO_2_ to the ocean interior through gravitational sinking of particles (Guidi et al., 2009). The role of phytoplankton in the ecosystem is further complicated due to the presence of mixotrophy. In addition to the traditional view of nonphagotrophic (i.e., purely phototrophic) phytoplankton (notably diatoms), there are phytoplanktonic taxa capable of phagotrophy of bacteria and small protists (called constitutive mixotrophs), as well as phytoplanktonic species living in symbiosis with a heterotrophic host which is defined as endosymbiotic specialist nonconstitutive mixotroph (Mitra et al., 2016). The remaining cases of mixotrophy correspond to those heterotrophic organisms that can temporarily retain functional chloroplasts from their ingested algal preys (kleptoplastidy) (Mitra et al., 2016).

Genetic surveys of the structure and composition of microbial communities are typically performed by PCR amplification and sequencing of a fragment of the small subunit of the rRNA gene from an environmental sample (rRNA gene metabarcoding). The fraction of the obtained sequencing reads corresponding to a given taxon is then used as a proxy for its relative abundance. Most studies have so far focused on taxonomically informative fragments of the hypervariable regions of the 16S (prokaryote and chloroplast) or 18S (eukaryotic nuclear) rRNA genes that are by far the most represented in reference databases (Guillou et al., 2013; Pawlowski et al., 2012; Quast et al., 2013). These markers are occasionally targeted in both DNA and RNA to exclude inactive microbes and as proxies of metabolic activities (Campbell et al., 2011; Logares et al., 2012), but more recent studies have indicated severe limitations of this concept and only mRNA can be considered as an indicator of the metabolic state (Blazewicz et al., 2013).

Although rRNA gene metabarcoding is widely used, it has limitations (in addition to the error sources during DNA extraction or sequencing that also affect other molecular methods). Firstly, PCR amplification bias due to mismatches of universal primers on the target sites of certain taxa can generate differences between the observed and the genuine relative read abundances as large as 10‐fold, either when using the 16S (Parada et al., 2016; Polz & Cavanaugh, 1998; Wear et al., 2018) or 18S rRNA gene markers (Bradley et al., 2016). Shotgun sequencing is a PCR‐free alternative and consists of the detection of these marker genes in metagenomes (Liu et al., 2007; Logares et al., 2014; Obiol et al., 2020) or in total RNA metatranscriptomes (Urich et al., 2008) (given that rRNA comprises >85% of total RNA in most organisms).

Another limitation of rRNA‐based approaches is that the copy‐number of these marker genes varies greatly among species. While bacterial genomes contain between one and 15 copies of the 16S rRNA gene (Acinas et al., 2004; Kembel et al., 2012; Větrovský & Baldrian, 2013), protists can differ by >5 orders of magnitude in their 18S rRNA gene copy numbers, from 33,000 in dinoflagellates to one in small chlorophytes (Godhe et al., 2008; Mäki et al., 2017; de Vargas et al., 2015; Zhu et al., 2005). Due to a positive association between rRNA gene copy number and cell size, it was proposed that the rRNA gene metabarcoding reads reflect the relative biovolume proportion for a given taxon (Lamb et al., 2019). Biovolume is a proxy of biomass, which is a relevant variable for studies of energy and matter fluxes such as food web structures and biogeochemical cycles. However, there is still little consensus for use of the rRNA gene as a biovolume estimator due to the poor correlations reported in many studies (Lamb et al., 2019; Lavrinienko et al., 2021; van der Loos & Nijland, 2021; Santoferrara, 2019). Instead, there have been attempts to infer relative cell abundances from rRNA gene metabarcoding by correcting the copy number variation. Although the copy number remains unknown for most microbial species, its assessment in different organisms could lead to the establishment of correction factors by assuming that the copy number is phylogenetically conserved. These approaches were applied to the 16S rRNA gene in bacteria, but accuracy is limited for taxa with no close representatives in reference phylogenies (Kembel et al., 2012; Louca et al., 2018; Starke et al., 2020). In protists, this correction is even more challenging due to intraspecies variation in 18S rRNA gene copy number. For example, it varies almost 10‐fold among 14 different strains of the haptophyte *Emiliana huxleyi* (Gong & Marchetti, 2019). In addition, there are major difficulties for generating a comprehensive database of 18S rRNA copy numbers (Gong & Marchetti, 2019).

Finally, assigning functional traits such as photosynthesis based solely on rRNA genes (or other housekeeping markers) is challenging and limited to what we know from experts and the literature. Indeed, while photosynthesis occurs in almost all cyanobacteria (except a few symbiotic lineages that have lost it [Nakayama et al., 2014; Thompson et al., 2012]), it is not necessarily conserved within protist taxa, such as dinoflagellates, of which only around half of known species are photosynthetic (Dorrell & Smith, 2011; Saldarriaga et al., 2001), chrysophytes (Dorrell et al., 2019; Dorrell & Smith, 2011) and chrompodellids (the sister lineage of apicomplexan parasites) (Janouškovec et al., 2015). This is an important issue because we still do not know how extended among related lineages are the independent events of chloroplast gains and losses or the extent of loss of photosynthesis with retention of the plastids. Thus, it is not possible to annotate the photosynthesis trait to those sequences whose taxonomic affiliation is, for example, “unknown dinoflagellate”.

The whole phytoplankton community covering both cyanobacteria and eukaryotic phytoplankton can be achieved by combining the two different rRNA marker genes (McNichol et al., 2021; Needham et al., 2018; Urich et al., 2008; Yeh et al., 2021). Alternatively, this can be directly carried out by targeting the plastidial and cyanobacterial versions of the 16S rRNA gene (Fuller, Campbell, et al., 2006; Fuller, Tarran, et al., 2006; Kirkham et al., 2011, 2013; Lepère et al., 2009; McDonald et al., 2007; Shi et al., 2011). However, dinoflagellates and chrompodellids are not represented in these surveys because their plastidial 16S rRNA genes are extremely divergent (Green, 2011), and this approach can still capture nonphotosynthetic plastids and kleptoplastids (functional plastids temporarily retained from ingested algal prey). It should be noted that kleptoplastid‐bearing species can still be major primary producers such as in cases of red tide ciliates (Johnson, 2011). Plastid‐encoded markers directly involved in photosynthesis have also been used, such as *psbA* and *rbcL* (Man‐Aharonovich et al., 2010; Paul et al., 2000; Zeidner et al., 2003). The *psbA* gene encodes the D1 protein of photosystem II and is also found in cyanophages (viruses) and the used primers target essentially the cyanobacterial and cyanophage sequences (Adriaenssens & Cowan, 2014). The *rbcL* gene encodes the large subunit of the ribulose‐l,5‐diphosphate carboxylase/oxygenase (RuBisCO). There are multiple *rbcL* types, even in nonphotosynthetic organisms, and the gene location varies: form I is plastid‐encoded in plants and most photosynthetic protists (and is present in cyanobacteria) while form II is nuclear‐encoded in peridinin dinoflagellates and chrompodellids (and is also present in proteobacteria) (Tabita et al., 2008). The different *rbcL* variants thus prevent its use for covering the whole phytoplankton community.

Plastid‐encoded genes (16S rRNA, *psbA*, *rbcL*) are affected by copy number variability among taxa not only at the level of gene copies (for example, four 16S rRNA gene copies in the plastid genome of the euglenophyte *Euglena gracilis* and six in the prasinophyte *Pedinomonas minor* [Decelle et al., 2015]), but also at the level of plastid genomes per plastid, and plastids per cell. The plastid number per cell varies from one or a few in most microalgal species to more than 100 in many centric diatoms (Decelle et al., 2015). In addition, it varies according to biotic interactions, for example, the haptophyte *Phaeocystis* has two plastids in a free‐living stage but increases up to 30 when present as an endosymbiont of radiolarians (Decelle et al., 2019). Photosynthetic eukaryotes typically maintain 50–100 plastid genome copies per plastid, but there is a continuous increase throughout development and during cell cycle progression (Armbrust, 1998; Coleman & Nerozzi, 1999; Hiramatsu et al., 2006; Koumandou & Howe, 2007; Oldenburg & Bendich, 2004). These limitations of plastid‐encoded marker genes can be circumvented by the use of photosynthetic nuclear‐encoded genes, which is still an unexplored approach.

In spite of the aforementioned biases, gene metabarcoding either based on rRNA genes or on alternative marker genes such as *psbA* or *rbcL* usually assume that the relative abundance of the gene sequences is an accurate measure of the relative abundance of the organisms containing those sequences. However, this assumption can lead to misleading inferences about microbial community structure and diversity, including relative abundance distributions, estimates of the abundance of different taxa, and overall measures of community diversity and similarity (Bachy et al., 2013; Egge et al., 2013; Kembel et al., 2012; Mäki et al., 2017; Medinger et al., 2010; Pinto & Raskin, 2012). For example, less than 30% of the variance in true organismal abundance is explained by observed prokaryotic 16S rRNA gene abundance in some simulation analyses (Kembel et al., 2012). In addition, comparative studies between morphological and molecular approaches in environmental samples or in mock communities revealed discrepancies up to several orders of magnitude among protist taxa with regard to their relative abundances (Bachy et al., 2013; Egge et al., 2013; Mäki et al., 2017; Medinger et al., 2010; Pawlowski et al., 2016). Most of these studies focused on the biases generated by primers and copy‐number variations, but not on uncertainties in assigning photosynthetic potential (e.g., differentiating between functionally photosynthetic and secondarily nonphotosynthetic species).

We deemed it important to find more accurate alternative procedures to the most widely‐used molecular approaches to make reliable estimations of species abundance, an important measure for inferring community assembly processes. We propose here to target nuclear‐encoded single‐copy core photosynthetic genes obtained from metagenomes to circumvent these limitations: the method is PCR‐free, and the genes are present in both prokaryotes and eukaryotes, in one copy per genome. We focused on the *psbO* gene, which encodes the manganese‐stabilising polypeptide of the photosystem II oxygen evolving complex. It is essential for photosynthetic activity and has the additional advantage of lacking any non‐photosynthetic homologues. We applied and validated this new strategy with the *Tara* Oceans data sets (Table 1). We quantified the biases in taxon abundance estimates using rRNA gene markers as compared to optical approaches (flow cytometry, microscopy), and we compared these patterns with those obtained by our proposed method. We also searched for *psbO* within metatranscriptomes to analyse its potential use as a proxy of photosynthetic activity and/or biovolume (due to the higher transcript level requirements of larger cells). Besides finding a more relevant marker gene for phytoplankton, we also propose its combination with single‐copy housekeeping genes (e.g., *recA* for bacteria and genes encoding ribosomal proteins in eukaryotes) to estimate the fraction of photosynthetic members in the whole community or in a given taxon. Finally, we show how the approach improves measures of microbial community diversity, structure, and composition as compared to rRNA gene metabarcoding.

**TABLE 1:** *Tara* Oceans data sets relevant to the current study

Target	Size fraction	Data set	Data set construction	Subset used in the current study	References and link
Prokaryotes and picoeukaryotes	0.2–3 µm	16S miTags (metagenomic Illumina tags)	16S rRNA gene sequences were identified in metagenomes and assembled. OTUs were defined at 97% identity cutoff	726 OTUs assigned to picophytoplankton (258 cyanobacteria +468 eukaryotic phytoplankton)	Salazar et al., 2019 https://www.ocean‐microbiome.org/
Eukaryotes	Five size fractions (0.8–2,000 µm)^a^	18S rRNA gene (V9 region) metabarcoding	PCR amplification of the V9 region (~130 base pairs length) of 18S rRNA gene followed by the high‐throughput sequencing of the amplicons, which were clustered into OTUs using SWARM	31,930 OTUs assigned to eukaryotic phytoplankton (including photosynthetic dinoflagellates and chrysophytes)	de Vargas et al., 2015; Ibarbalz et al., 2019 https://zenodo.org/record/3768510#.Xraby6gzY2w
Eukaryotes	Five size fractions (0.8–2,000 µm)^a^	18S miTags (metagenomic Illumina tags)	18S rRNA gene reads were identified in metagenomes and annotated against PR2 reference database	~20 M unique reads assigned to eukaryotic phytoplankton (including photosynthetic dinoflagellates and chrysophytes)	This study https://www.ebi.ac.uk/biostudies/studies/S‐BSST762
Prokaryotes and picoeukaryotes	0.2–3 µm	Ocean Microbial Reference Gene Catalogue (OM‐RGC‐v2)	Unigenes assembled from metagenomes and clustered at 95% identity. Metagenomic and metatranscriptomic reads were then mapped on these unigenes.	307 *psbO* sequences from cyanobacteria and eukaryotic phytoplankton	Salazar et al., 2019 https://www.ocean‐microbiome.org/
Eukaryotes	Five size fractions (0.8–2,000 µm)^a^	Marine Atlas of *Tara* Oceans Unigenes (MATOU‐v1)	Transcribed sequences assembled from poly‐A+ metatranscriptomes and clustered at 95% identity. Metagenomic and metatranscriptomic reads were then mapped on these unigenes.	10,646 *psbO* sequences from eukaryotic phytoplankton	Carradec et al., 2018 http://www.genoscope.cns.fr/tara/
Prokaryotes and eukaryotes	Six size fractions (0.2–2,000 µm)^b^	Metagenomes	Raw metagenomic reads	~3.2 million metagenomic reads aligned to a curated database of *psbO* sequences	EBI accessions: PRJEB1787 PRJEB1788 PRJEB4352 PRJEB4419 PRJEB9691 PRJEB9740 PRJEB9742
Prokaryotes and eukaryotes	<200 µm	Flow cytometry		Abundances and biovolume of picocyanobacteria and eukaryotic picophytoplankton	Hingamp et al., 2013; Gasol & Morán, 2015; this study https://data.mendeley.com/datasets/p9r9wttjkm/2 https://www.ebi.ac.uk/biostudies/studies/S‐BSST761
Eukaryotes	5–20 µm	Confocal microscopy		Abundance and biovolume of nanophytoplankton	Colin et al., 2017 https://www.ebi.ac.uk/biostudies/studies/S‐BSST51
Eukaryotes	20–180 µm	Light microscopy		Abundance of microphytoplankton	Malviya et al., 2016; this study https://www.ebi.ac.uk/biostudies/studiesS‐BSST761
Prokaryotes	20–180 µm	Confocal microscopy		Abundance of the symbiotic cyanobacteria *Richelia*/*Calothrix* and the colony‐forming *Trichodesmium*	Pierella Karlusich et al., 2021 https://static‐content.springer.com/esm/art%3A10.1038%2Fs41467‐021‐24299‐y/MediaObjects/41467_2021_24299_MOESM11_ESM.xlsx

^a^ 0.8–5 µm, 5–20 µm, 20–180 µm, 180–2000 µm.

^b^ 0.2–3 µm, 0.8–5 µm, 5–20 µm, 20–180 µm, 180–2000 µm.

## 2 MATERIALS AND METHODS

### 2.1 Search for phytoplankton marker genes

To estimate cell‐based relative abundances of the major marine phytoplankton groups, we searched for genes present in all photosynthetic organisms (both prokaryotes and eukaryotes) and with low copy‐number variability among taxa. To fulfil the latter requirement, we first excluded plastid‐encoded genes to avoid the variations in number of chloroplasts per cell and in number of chloroplast genomes per organelle. We did this by retrieving sequences from the KEGG (Kanehisa, 2000) database that are assigned to the photosynthetic electron transport chain, the Calvin Cycle and chlorophyll biosynthesis, to be used as queries for sequence similarity searches against >4,100 plastid genomes available at NCBI (https://www.ncbi.nlm.nih.gov/genome/organelle/). For this, blast version 2.2.31 (“tBLASTn” program) searches were conducted with an e‐value cutoff of 1e‐20 (Camacho et al., 2009). To retain only core photosynthetic genes, that is, those present in all phototrophs, we then made an equivalent BLAST search against cyanobacterial and eukaryotic nuclear genomes from the IMG (Chen et al., 2019) and PhycoCosm (Grigoriev et al., 2021) databases and from the polyA‐derived transcriptomes of the Marine Microbial Eukaryote Transcriptome Sequencing Project (MMETSP) (Keeling et al., 2014). To minimize false‐negative cases, only completely sequenced genomes were considered for establishing gene absence. This survey was also used for determining gene copy number variation.

This survey resulted in a list of five genes that are core, nuclear‐encoded and present in low copy numbers (Table 2). For selecting a gene marker of phytoplankton among them, we carried out a deeper sequence analysis to detect non‐photosynthetic homologues and to see if the phylogeny reflects the evolutionary history of cyanobacteria and endosymbiosis. We first performed a sequence similarity search using HMMer version 3.2.1 with gathering threshold option (http://hmmer.org/) for the corresponding Pfam domain against the translated sequenced genomes and transcriptomes from PhycoCosm and MMETSP as well as in the whole IMG database (including viruses, archaea, bacteria and nonphotosynthetic eukaryotes). The Pfams used in the search were: MSP (PF01716) for PsbO, Rieske (PF00355) for PetC, PRK (PF00485) for phosphoribulokinase, UbiA (PF01040) for chlorophyll‐*a* synthase, and NAD_binding_1 (PF00175) for ferredoxin:NADP^+^ reductase. cdhit version 4.6.4 (Li & Godzik, 2006) was used at an 80% identity cutoff to reduce redundancy. These sequences were used for building a protein similarity network using EFI‐EST tool (Zallot et al., 2019) and Cytoscape visualization (Shannon et al., 2003), and BlastKOALA with default parameters for functional annotation (Kanehisa et al., 2016). These analyses led us to focus on *psbO* as a gene marker for phytoplankton, for which we did a deeper analysis by building its phylogeny in the following way. Protein sequences were aligned with mafft version 6 using the G‐INS‐I strategy (Katoh & Toh, 2008). Phylogenetic trees were generated with PhyML version 3.0 using the LG substitution model plus gamma‐distributed rates and four substitution rate categories (Guindon et al., 2010). The starting tree was a BIONJ tree and the type of tree improvement was subtree pruning and regrafting. Branch support was calculated using the approximate likelihood ratio test (aLRT) with a Shimodaira–Hasegawa‐like (SH‐like) procedure.

**TABLE 2:** List of nuclear‐encoded photosynthetic genes present in all cyanobacteria and eukaryotic phytoplankton. These genes are always nuclear‐encoded, with the exception of amoeba from the genus *Paulinella* (Figure 2a), which has gained its plastid only very recently and independently of the event at the origin of all other known plastids (Singer et al., 2017; Yoon et al., 2006)

Gene	Pathway	Function	Copies	Nonphotosynthetic homologues	References
*prk* (phosphoribulokinase)	Calvin‐Benson‐Bassham cycle	phosphorylation of ribulose−5‐phosphate to ribulose−1,5‐bisphosphate, the RuBisCO substrate	1	PRKs from archaea and bacteria	Jaffe et al. (2019); Kono et al. (2017)
*chlG* (chlorophyll‐*a* synthase)	Chlorophyll‐*a* biosynthesis	last step of chlorophyll‐*a* biosynthesis	1	Prenyltransferases with UbiA domain	Wang et al. (2015)
*petH* (ferredoxin‐NADP^+^ oxidoreductase)	Photosynthetic electron transport chain	last step of the linear electron flow (NADP^+^ reduction by ferredoxin or flavodoxin)	1–3	‐FNRs involved in nitrogen metabolism ‐FNRs from nonphotosynthetic plastids ‐C‐terminal region of benzoyl‐CoA oxygenase component A (BoxA) from bacteria	Pierella Karlusich and Carrillo (2017); Mohamed et al. (2001)
*petC*	Photosynthetic electron transport chain	Rieske subunit of the chloroplast Cyt *b*_6_ *f* complex	2–3	Rieske proteins from mitochondria, bacteria and archaea	Lebrun et al. (2006); Veit et al. (2016)
*psbO*	Photosynthetic electron transport chain	Manganese‐stabilizing protein of photosystem II	1–2	No	Pierella Karlusich et al. (2015)

### 2.2 Analysis of *Tara* Oceans data sets

*Tara* Oceans expeditions between 2009 and 2013 performed a worldwide sampling of plankton in the upper layers of the ocean (Sunagawa et al., 2020). To capture the whole size spectrum of plankton, a combination of filter membranes with different pore sizes (size‐fractionation) was used to separate organisms by body size (Pesant et al., 2015). There is an inverse logarithmic relationship between plankton size and abundance (Belgrano et al., 2002; Pesant et al., 2015), so small size fractions represent the numerically dominant organisms in terms of cell abundance (albeit not necessarily in terms of total biovolume or biomass). Thus, the protocols consisted in the filtering of higher seawater volumes for the larger size fractions (Pesant et al., 2015). Five major organismal size fractions were collected: picoplankton (0.2–3 μm size fraction), piconanoplankton (0.8–5 μm size fraction), nanoplankton (5–20 μm size fraction), microplankton (20 to 180 μm), and mesoplankton (180 to 2,000 μm). These plankton samples were leveraged to generate different molecular and optical data sets that were analysed in the current study (Table 1). We exclusively used the data sets corresponding to surface samples (5 m depth).

### 2.3 *psbO*‐based community data

To use metagenomic and metatranscriptomic read abundances of *psbO* as a proxy of phytoplankton relative cell abundance and “activity”, respectively, we carried out an HMMer search as stated in the previous section against the two *Tara* Oceans gene catalogues: the Ocean Microbial Reference Gene Catalogue version 2 (OM‐RGC.v2) covering prokaryotic and eukaryotic picoplankton (<3 µm), and the Marine Atlas of *Tara* Oceans Unigenes version 1 (matou.v1) covering eukaryotic plankton ranging from 0.8 to 2,000 μm (Table 1). The metagenomic and metatranscriptomic reads were already mapped onto both catalogues, thus we retrieved these values for those sequences obtained by our HMMer search. For the taxonomic assignment of *psbO* unigenes, we performed a phylogenetic placement of the translated sequences on the PsbO protein reference phylogenetic tree described in the previous section. A set of 50 unigenes were translated and the PsbO specific Pfam PF01716 region was retrieved for the analysis in the following way. First, they were aligned against the reference alignment described in the previous section using the option ‐‐add of mafft version 6 with the G‐INS‐I strategy (Katoh & Toh, 2008). The resulting alignment was used for building a phylogeny with PhyML version 3.0 as described above (Guindon et al., 2010). The sequences were classified using the APE library in R (Paradis & Schliep, 2019) according to their grouping in monophyletic branches of statistical support >0.7 with reference sequences of the same taxonomic group.

Due to challenges of assembling eukaryotic genomes from complex metagenomes, the MATOU‐v1 catalogue only contains sequences assembled from poly‐A‐tailed RNA (Alberti et al., 2017; Carradec et al., 2018), which biases against prokaryotic sequences. To determine the structure of the whole phytoplankton community (including both cyanobacteria and eukaryotic phytoplankton), we aligned all the metagenomic reads from *Tara* Oceans to a curated database of *psbO* sequences (described below; see also Table 1). The analysis was carried out using the bwa tool version 0.7.4 (Li & Durbin, 2009) with the following parameters: ‐minReadSize 70 ‐identity 80 ‐alignment 80 ‐complexityPercent 75 ‐complexityNumber 30. Abundance values were expressed in rpkm (reads per kilobase covered per million of mapped reads).

In general, the rpkm values for the different taxa under study were converted to percentage of (either total or eukaryotic) phytoplankton. However, for a specific analysis the *psbO* rpkm values were normalized by those values from single‐copy housekeeping genes: by bacterial *recA* (Sunagawa et al., 2013) to estimate the contribution of cyanobacteria in the bacterioplankton, or by the average abundance of 25 genes encoding ribosomal proteins (Carradec et al., 2018; Ciccarelli et al., 2006) to estimate the contribution of phytoplankton among eukaryotes. The abundance values for *recA* were retrieved from a previous study (Pierella Karlusich et al., 2021) while the ribosomal proteins were recovered from the MATOU‐v1 and OMRGC‐v2 abundance tables.

### 2.4 rRNA gene‐based community data

We used three different data sets generated by *Tara* Oceans for “traditional” DNA‐based methods: 16S rRNA gene miTags (metagenomic Illumina tags, i.e., 16S rRNA gene assemblies derived from metagenomes sequenced with an Illumina platform) for size fraction 0.2–3 µm and 18S rRNA gene miTags and 18S rRNA gene (V9 region) metabarcoding for sizes fractions 0.8–5, 5,20, 20–180, 180–2,000 µm (Table 1). We extracted the relative abundances for the 726 operational taxonomic units (OTUs) assigned to picophytoplankton (cyanobacteria and chloroplasts) from the 16S miTags and the 31,930 OTUs assigned to eukaryotic phytoplankton from the V9‐18S metabarcoding data. In addition, we generated 18S rRNA miTags in the following way. We extracted metagenomic reads for rRNA using SortMeRNA (Kopylova et al., 2012) and those with ≥100 bp length with no Ns were dereplicated at the study level using vsearch v2.18 (Rognes et al., 2016). The resulting unique sequences were pairwise compared to PR2 v4.14 (Guillou et al., 2013) using the vsearch's command ‐‐usearch_global to find the best hit defined as the reference sequence with the least differences in the region covering 100% of the query sequence. Only hits with ≥80% identity were kept. Each unique sequence inherits the taxonomy of the best hit and, in case of ties, the last common ancestor of the reference sequences is used. The read abundances were expressed as relative abundance (%) in relation to the picophytoplankton community for 16S miTags, and in relation to eukaryotic phytoplankton for V9‐18S metabarcoding and 18S miTags.

The assignations of the 16S and 18S rRNA sequences to phytoplankton were based on literature and expert information and included photosynthetic dinoflagellates and chrysophytes when their taxonomic resolution was sufficient to match known photosynthetic lineages. A full description of the 18S taxonomic classification procedure is at http://taraoceans.sb‐roscoff.fr/EukDiv/ and the last version of the trait reference database used in the current study is available at https://zenodo.org/record/3768951#.YcULlHVKisx. In the case of 16S miTags, the taxonomic assignment was improved by building a phylogenetic tree with the 16S miTags sequences and a curated set of references from NCBI and MMETSP. Sequences were aligned using mafft v7.0 (Katoh & Standley, 2013) with ‐‐auto setting option and then trimmed using trimal with the ‐gt 0.5 and ‐gt 0.8 settings, and the resulted alignment was used for tree building using RAxML v8 (Stamatakis, 2014) (100 bootstrap replicates, GTRCAT substitution model).

### 2.5 Optical‐based community data

We also used quantitative optical data generated by *Tara* Oceans (Table 1), where cell abundance is assumed to be more accurate and less biased, and additional features such as biovolume can be determined. The data sets cover: flow cytometry for picoplankton, confocal microscopy for 5–20 μm size fraction, and light microscopy for 20–180 µm size fraction.

Flow cytometry counts were determined on three 1 ml seawater samples filtered through 200 μm that were fixed with cold 25% glutaraldehyde (final concentration 0.125%) and stored at –80°C until analysis. Details about the procedure can be found in (Gasol & Morán, 2015; Hingamp et al., 2013; Pierella Karlusich et al., 2021). The cell biovolume was calculated using the equation of (Calvo‐Díaz & Morán, 2006) on the bead‐standardized side scatter of the populations and considering cells to be spherical.

Quantitative confocal microscopy was performed using environmental High Content Fluorescence Microscopy (eHCFM) (Colin et al., 2017). Briefly, samples were fixed with 10% monomeric formaldehyde (1% final concentration) buffered at pH 7.5 and 500 μl EM grade glutaraldehyde (0.25% final concentration) and kept at 4°C until analysis. Sample collection, preparation, and imaging acquisition is described in (Colin et al., 2017). The 5–20 μm size fraction has been classified at a coarse taxonomic level (with an estimated accuracy of 93.8% at the phylum or class level), into diatoms, dinoflagellates, haptophytes, and other/unclassified eukaryotic phytoplankton (Colin et al., 2017). We used the major and minor axis of every image to calculate their ellipsoidal equivalent biovolume. The 20–180 µm size fraction is also available, but the curated taxonomic annotation is limited to symbiotic (*Richelia*, *Calothrix*) and colony‐forming (*Trichodesmium*) nitrogen‐fixing cyanobacteria (Pierella Karlusich et al., 2021), which were also used in the current study.

For light microscopy, three ml of each sample (from 20–180 µm size fractions) were placed in an Utermöhl chamber with a drop of calcofluor dye (1:100,000) which stains cellulose, thus allowing to better detect and identify dinoflagellates. Cells falling in two or four transects of the chamber were identified and enumerated using an inverted light microscope (Carl Zeiss Axiophot200) at 400x magnification.

To be compared with the molecular data, the optical data were expressed as relative abundance (%). In the case of flow cytometry, the relative abundance is calculated over the total number of cells counted as picophytoplankton (*Prochlocococcus* + *Synechococcus* + eukaryotic picophytoplankton). In the case of confocal and optical microscopy, the values are expressed as percentage of total eukaryotic phytoplankton cells.

### 2.6 *psbO* database generation

We compiled, curated and annotated a database of >18,000 unique *psbO* sequences covering cyanobacteria, photosynthetic protists, macroalgae and land plants (Figure S1). It includes sequences retrieved from IMG, NCBI, MMETSP and other sequenced genomes and transcriptomes from cultured isolates, as well as from the environmental sequence catalogues from Global Ocean Sampling (Rusch et al., 2007) and *Tara* Oceans (Carradec et al., 2018; Delmont et al., 2020, 2021; Salazar et al., 2019). The taxonomic assignment of environmental sequences of *psbO* was determined by the placement of their translated sequences on a PsbO protein reference phylogeny as described in the previous section. The database can be downloaded from the EMBL‐EBI repository BioStudies (www.ebi.ac.uk/biostudies) under accession S‐BSST659. We expect to maintain it updated to facilitate its incorporation in molecular‐based surveys.

### 2.7 Plotting and statistical analysis

Graphical analyses were carried out in R language (http://www.r‐project.org/) using *ggplot2* (Wickham, 2016) and treemaps were generated with *treemap*. Maps were generated with *borders* function in *ggplot2* and *geom_point* function for bubbles or *scatterpie* package for pie charts (Yu, 2018). Spearman's Rho correlation coefficients and *p*‐values were calculated using the *cor.test* function of the *stats* package. Shannon diversity indexes were calculated using the *vegan* package (Oksanen et al., 2020). Intra‐ and interspecific genetic distances were calculated in megax (Kumar et al., 2018) using the maximum composite likelihood model.

## 3 RESULTS

### 3.1 Search for phytoplankton marker genes

**FIGURE 1:**
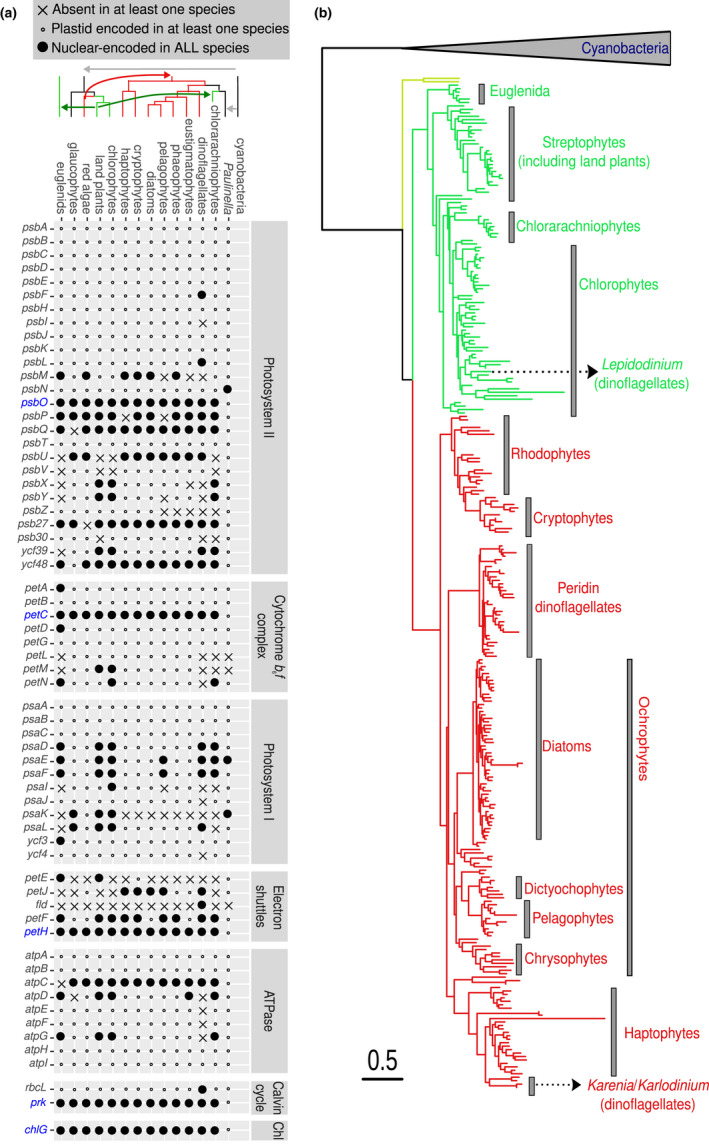
Identification of nuclear‐encoded core photosynthetic gene marker candidates. (a) Presence and location of the genes encoding proteins involved in photosynthesis. The evolutionary relationship between the analysed lineages is represented at the top of the panel, with the arrows indicating the different endosymbiosis events. The genes found to be core and nuclear‐encoded are indicated in blue. The only exception is the amoeba from the genus *Paulinella*, which has gained its plastid very recently and independently of the event at the origin of all other known plastids, thus still retaining these genes in its plastid genome (Singer et al., 2017; Yoon et al., 2006). (b) Phylogeny of PsbO protein. Translated sequences from genomes and transcriptomes of cultured phytoplankton species were used for the phylogeny reconstruction. The scale bar indicates the number of expected amino acid substitutions per site per unit of branch length

We first analysed transcriptomes and nuclear and plastid genomes derived from cultured strains to inventory photosynthetic genes in relation to their genome location (nuclear‐ vs. plastid‐encoded) and taxonomic prevalence (core vs. noncore, i.e., present in all phototrophs or not) (see Methods; Figure 1a). Among the plastid‐encoded genes, we identified phytoplankton marker genes previously used in environmental surveys, such as *psbA* (Man‐Aharonovich et al., 2010; Zeidner et al., 2003), *rbcL* (nuclear encoded in dinoflagellates containing peridinin) (Paul et al., 2000) and *petB* (Farrant et al., 2016) (Figure 1a).

Among the nuclear‐encoded genes, we retrieved some which are noncore, such as those encoding flavodoxin (*fld*) and plastocyanin (*petE*), but also five core genes (Figure 1a; Table 2). These five genes are present in low‐copy number and encode components of the photosynthetic electron transport chain (*psbO*, *petC* and *petH*), the carbon fixation pathway (*prk*) or chlorophyll biosynthesis (*chlG*) (Table 2). The absence of nonphotosynthetic homologues is a unique characteristic of *psbO* (Table 2 and Figures S2–S6), reflecting its essential role in the photosynthetic oxygen evolution reaction (i.e., the splitting of a water molecule into its protons and electrons using the energy of light, generating free oxygen as a byproduct), and a clear advantage for its use as a marker gene for phytoplankton. Previous studies of secondarily nonphotosynthetic eukaryotes have marked its presence or absence as being an effectively universal predictor of photosynthetic potential (Dorrell et al., 2019). Its phylogeny additionally reflects the evolutionary history of endosymbiosis (Figure 1b; Pierella Karlusich et al., 2015), with few or no post‐endosymbiotic horizontal replacements known, so we focused on this gene for the analysis of environmental samples.

Although no global barcoding gap (i.e., a distance threshold set for all species) was detected when checking intra‐ versus interspecific divergences for eukaryotic phytoplankton based on *psbO*, it was neither observed with the V9 region of the traditional marker 18S rRNA gene (Figure S7). This absence does not necessarily preclude specimen identification, which relies upon the presence of a “local“ barcoding gap (i.e., a query sequence being closer to a conspecific sequence than a different species), rather than the “global” barcoding gap (i.e., a distance threshold set for all species) that is required for species discovery (Collins & Cruickshank, 2013).

We retrieved the *psbO* sequences from the two *Tara* Oceans gene catalogues (the picoplankton catalogue OM‐RGC.v2 and the eukaryotic catalogue MATOU.v1; see Methods and Table 1). A total of 307 distinct sequences were identified in OM‐RGC.v2 (202 from *Prochlorococcus*, 79 from *Synechoccocus* and 26 from eukaryotic picophytoplankton), with an average length for the conserved coding region of 473 base pairs (bp) and a range between 94 and 733 bp. A total of 10,646 sequences from eukaryotic phytoplankton were retrieved from MATOU.v1, with an average length for the conserved coding region of 385 bp and a range between 66 and 784 bp. The analyses of the metagenomic and metatranscriptomic read abundances of these sequences are presented in the following sections.

### 3.2 Marine phytoplankton community structure based on *psbO* shows remarkable differences with the traditional molecular approaches

The abundance and diversity of phytoplankton was first examined in *Tara* Oceans samples by focusing on the traditional marker genes coding for the small subunit of rRNA (16S for prokaryotes and plastids, 18S for eukaryotes) in the different size‐fractionated samples. We focused exclusively on the phytoplankton signal of these data sets, despite the uncertainties in assigning photosynthesis capacity in groups such as dinoflagellates and chrysophytes (this is evaluated in one of the next sections).

**FIGURE 2:**
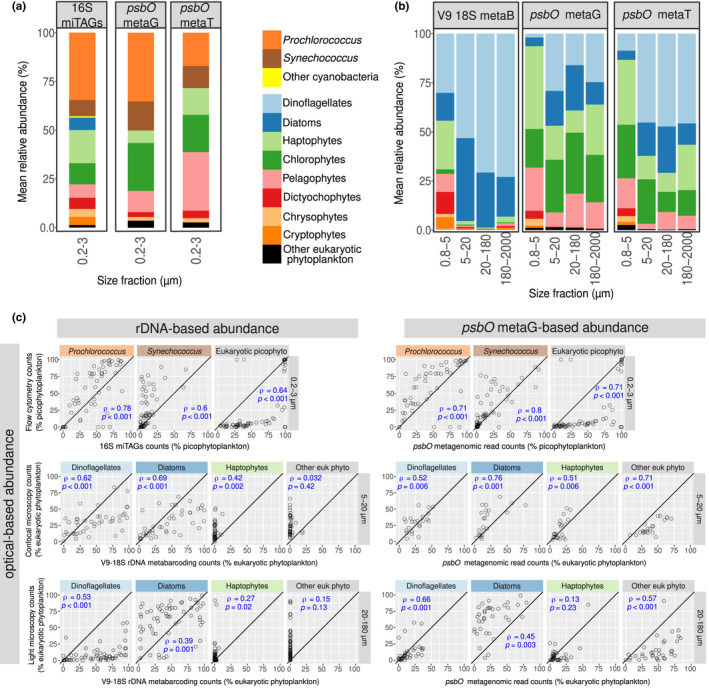
Congruence in relative abundances of the main phytoplankton groups based on different gene markers. (a–b) Average relative abundances for all surface samples of each size fraction using different marker genes. In (a), picocyanobacteria and eukaryotic picophytoplankton (0.2–3 μm) were analysed using 16S rRNA gene miTags and the metagenomic and metatranscriptomic read abundances for *psbO*. In (b), eukaryotic phytoplankton was analysed in larger size fractions using V9‐18S rRNA gene amplicons and the metagenomic and (polyA‐derived) metatranscriptomic *psbO* read abundances. (c) Correlations between relative abundances of different phytoplankton groups obtained with optical versus DNA‐based methodologies. In the upper panel, 16S rRNA gene miTags and *psbO*‐based relative abundances in picophytoplankton were compared with flow cytometry counts (values displayed as % total abundance of picophytoplankton). In the middle and lower panels, V9‐18S rRNA gene metabarcoding and metagenomic *psbO* relative abundances were compared with confocal microscopy counts from size fraction 5–20 μm and light microscopy counts from size fraction 20–180 µm (values displayed as % total abundance of eukaryotic phytoplankton). It is worth mentioning that the molecular and microscopy data were generated from the same samples, while there were differences between molecular data of 0.2–3 µm size fraction and flow cytometry data (see Methods). Axes are in the same scale and the diagonal line corresponds to a 1:1 slope. Spearman's Rho correlation coefficients and *p*‐values are displayed. The correlations of relative abundances between metatranscriptomic *psbO* reads and optical methods are shown in Figure S11

Based on 16S miTag read abundance among picophytoplankton (0.2–3 µm), the picocyanobacteria *Prochlorococcus* and *Synechococcus* were prevalent, while ~60% of the average read abundance was attributed to eukaryotic photosynthetic taxa such as haptophytes, chlorophytes, pelagophytes, dictyochophytes, chrysophytes, cryptophytes and diatoms (Figure 2a). In the larger size fractions, based on the V9‐18S region metabarcoding reads, diatoms and dinoflagellates were the most frequent among eukaryotic phototrophs, especially in the 5–20 μm and 20–180 μm size fractions (Figure 2b). In the 180–2000 μm fraction, diatoms and dinoflagellates were still abundant, due to the presence of large diameter cells (*Tripos*, *Pyrocystis*), chain‐forming (e.g., *Chaetoceros*, *Fragilariopsis*) or epizoic (e.g., *Pseudohimantidium*) species, without discarding that smaller species may be retained in samples of this size fraction due to net clogging or within herbivorous guts and faecal pellets. Relative abundance in the smaller 0.8–5 μm size fraction was much more homogeneously distributed between the different groups.

For *psbO*‐based methods, we found that metagenomic and metatranscriptomic reads from *Synechococcus*, *Prochlorococcus*, pelagophytes, chlorophytes and haptophytes were dominant among picophytoplankton (0.2–3 µm), along with dictyochophytes and chrysophytes (Figure 2a). In the larger size fractions, haptophytes, chlorophytes and pelagophytes clearly dominated the eukaryotic phytoplankton in the 0.8–5 μm size fraction, whereas diatoms and dinoflagellates were more abundant in the three larger size ranges (5–20 μm, 20–180 μm, 180–2,000 μm), although haptophytes, chlorophytes and pelagophytes were also detected in large quantities (Figure 2b). The potential cyanobacteria present in these large size fractions are presented later in another section due to the need to bypass the sequences assembled from poly‐A‐tailed RNA for analysing prokaryotes (see Methods and Table 1).

We noted some differences in *psbO* read counts between metagenomic and metatranscriptomic data sets. In the case of picophytoplankton, *Prochlorococcus* was enriched in metagenomes in comparison to (total RNA) metatranscriptomes (the small volume of *Prochlorococcus* probably constraints the transcript number per cell), the opposite was the case for pelagophytes and haptophytes, whereas no major changes were observed for *Synechococcus* and chlorophytes (Figure 2a and S8A). In the case of larger photosynthetic protists, dinoflagellates were highly abundant at the (polyA) transcript level in comparison to gene abundance (they probably have high gene expression levels because they predominantly perform post‐transcriptional regulation [Cohen et al., 2021; Roy et al., 2018]), the opposite was observed for pelagophytes and chlorophytes (in this latter taxon only in the 20–180 and 180–2,000 μm size ranges), whereas no major shifts were apparent for diatoms and haptophytes (Figure 2b and S8B).

The taxonomic abundance patterns based on *psbO* showed some differences with those from 16S miTags in the 0.2–3 µm size fraction, but exhibited remarkable differences with those based on V9‐18S metabarcoding from the large size fractions (Figure 2a–b and S9). When compared with the 16S miTags, no major changes were detected for *Prochlorococcus*, whereas the average *psbO* metagenomic contribution increased for *Synechococcus* (from ~8% to ~14%), at the expense of decreasing eukaryotic picoplankton contributions (from 57% to ~50%), which is expected due to the fact that the 16S rRNA is a plastid‐encoded gene in eukaryotes. When we compared *psbO* with V9‐18S metabarcoding, the differences were very significant. In the 0.8–5 μm size fraction, diatoms and dinoflagellates accounted for just ~6% of average *psbO* metagenomic read abundance but for ~44% of V9‐18S reads assigned to phytoplankton. In the three larger size ranges (5–20 μm, 20–180 μm and 180–2,000 μm), they accounted for 37%–47% of average *psbO* metagenomic read abundance, but for >90% of average V9‐18S read abundance. The V9‐18S read abundance was extremely low for haptophytes, chlorophytes and pelagophytes in these three size fractions (<7% average V9‐18S read abundance). When we compared the metatranscriptomic profile, it was more similar to the profile obtained with metagenomes than to that obtained with V9‐18S metabarcoding (Figure 2b).

### 3.3 Comparison with imaging data set indicates that *psbO* is a robust marker gene for estimating relative cell abundance of phytoplankton from metagenomes

To assess the accuracy of *psbO* gene counts for determining phytoplankton cell relative abundances, we carried out comparative analyses with imaging data sets. For the 0.2–3 µm size fraction, we compared relative abundances based on 16S and *psbO* counts with those inferred from flow cytometry (Figure 2c). Both genes were found to correlate well with flow cytometry. Although the correlations for eukaryotic picophytoplankton were strong (Spearman's Rho =0.64–0.71, *p*‐value <.001), the relationships were not linear and picoeukaryotes appeared at much higher relative abundances in metagenomes than in flow cytometry. This is consistent with the fact that flow cytometry can count cells of up to 10–20 µm diameter and was performed on seawater aliquots pre‐filtered through a 200‐μm mesh (see Methods), whereas DNA isolation of picoplankton was carried out on seawater volumes mainly filtered through 3 μm pore sizes. When we discarded eukaryotes to focus only on the ratio *Synechococcus* / (*Synechococcus* + *Prochlorococcus*) (Figure S10), flow cytometry data shows a linear relashiopship with *psbO* metagenomic reads, while 16S miTags reads underestimated *Synechococcus* and the opposite occurred for *psbO* metatranscriptomic reads. In addition, the highest correlation with flow cytometry data occurred with the *psbO* metagenomic counts (Spearman's Rho =0.92, 0.90 and 0.75, *p* <.001, for *psbO* metagenomic reads, *psbO* metatranscriptomic reads and 16S miTags, respectively).

The comparisons between microscopy and molecular data are direct as they were generated from the same size‐fractionated samples. For the 5–20 μm size fraction, the relative abundance of eukaryotic photosynthetic organisms was determined by cell counts using high‐throughput confocal microscopy. We compared these results with the proportions based on V9‐18S metabarcoding and *psbO* metagenomic reads (Figure 2d). The metabarcoding data for dinoflagellates and diatoms were in good agreement with the microscopy but it clearly underestimated the relative abundance of haptophytes and other eukaryotic phytoplankton. Regarding *psbO*, the metagenomic relative abundances were in stronger agreement with the microscopy counts for the four defined phytoplankton groups (Figure 2d). Therefore, in the 5–20 μm size fraction, diatoms and dinoflagellates displayed robust patterns of relative abundance using either V9‐18S metabarcoding or *psbO* metagenomic counts, while haptophytes and the other groups were better described by *psbO*.

In the 20–180 μm size fraction, the relative abundance of eukaryotic phytoplankton was determined by light microscopy. Again, the metabarcoding data for dinoflagellates and diatoms were in good agreement with the microscopy data but clearly underestimated the relative abundance of haptophytes and other eukaryotic phytoplankton groups (Figure 2d). The relative abundances of *psbO* metagenomic reads were in stronger agreement with the microscopy counts for the four defined phytoplankton groups, although the correlation with haptophytes was weaker (Figure 2d). Therefore, in the 20–180 μm size fraction, diatoms and dinoflagellates displayed robust patterns of relative abundance using either V9‐18S metabarcoding or *psbO* metagenomic counts, while haptophytes were weakly described by both methods and the other groups were much better described by *psbO*.

**FIGURE 3:**
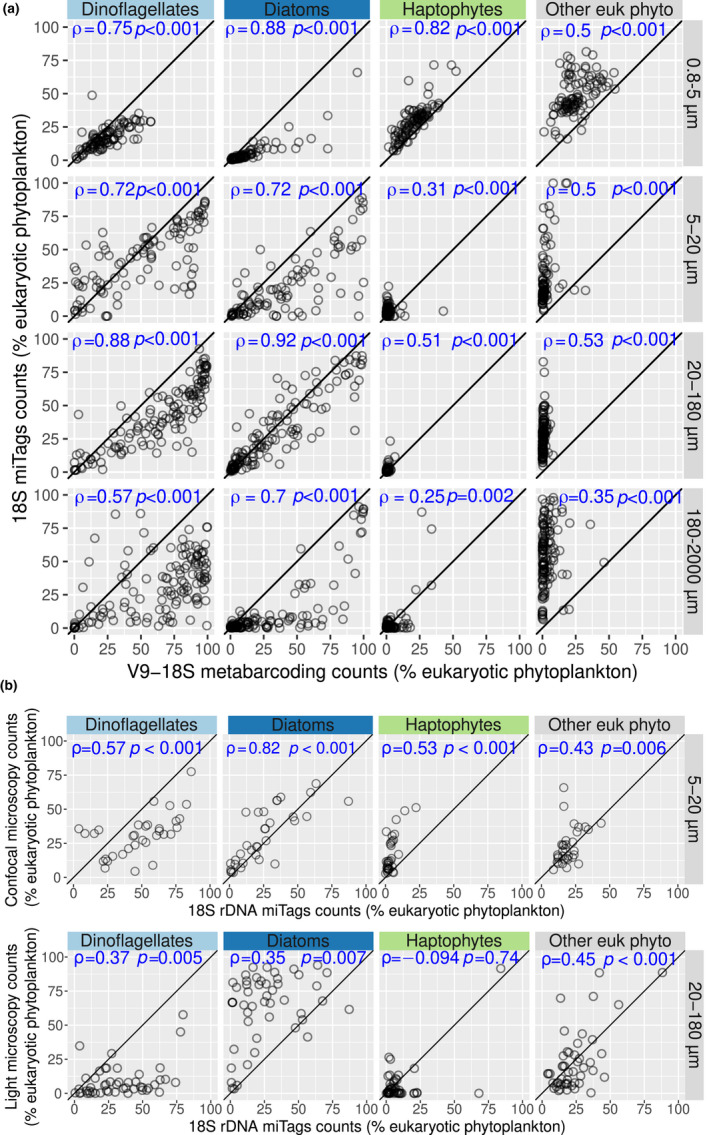
Comparison of the relative abundances for the main eukaryotic phytoplankton groups based on 18S rRNA gene miTags and other methodologies. (a) Correlations between relative read abundances between V9‐18S rRNA gene metabarcoding and 18S rRNA gene miTags for the main groups of eukaryotic phytoplankton from size‐fractionated samples. (b) Correlations between relative abundances of different eukaryotic phytoplankton groups obtained with microscopy versus 18S rRNA gene miTags. Spearman's correlation coefficients and *p*‐values are displayed in blue. Axis are in the same scale and the diagonal line corresponds to a 1:1 slope

The poor correlations between optical and rRNA data might be in part due to the type of metabarcodes (e.g., V9 fragments) and primers (e.g., 1389F/1392R), which have unique biases against certain taxa (McNichol et al., 2021). Therefore, we generated 18S miTags from the analysed metagenomes (see Methods) to disentangle the effect of PCR bias versus copy number in the patterns of V9‐18S metabarcoding. Our result showed that diatoms and dinoflagellates tend to be more abundant in V9‐18S metabarcoding than in 18S miTags, while the opposite occurs for haptophytes and even more so for the other phytoplankton groups (Figure 3a). When comparing the relative read abundances of 18S miTags against the microscopy counts, the correlations were much better than with the V9‐18S metabarcoding (Figure 3b). This suggests that PCR bias generates a strong effect in relative abundance estimations. Notwithstanding, the correlations against microscopy for 18S miTags are not as good as those for *psbO*, indicating that the copy number effect is indeed important (as well as the photosynthesis capacity annotation of the ribotypes, see below).

We also compared the relative abundances based on optical methods against those based on *psbO* metatranscriptomic reads, and in general we observed good agreement (Figure S11). Some phytoplankton groups displayed stronger correlations against optical methods using metatranscriptomic *psbO* counts than 16S miTAGs (e.g., *Synechococcus*) or V9‐18S metabarcoding (e.g., other eukaryotic phytoplankton in the 5–20 µm size fraction) (Figure 2d and S11). However, the consistency in relative abundance of *psbO* reads with optical methods was always better for metagenomes than for metatranscriptomes (Figure 2d and S11).

### 3.4 Comparison with optical‐based biovolume suggests that neither *psbO* nor rRNA genes are good proxies for estimating relative proportion of biovolume

**FIGURE 4:**
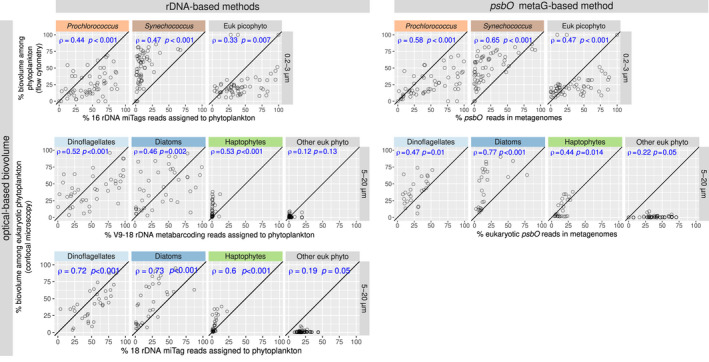
Correlation between relative biovolume (based on optical methods) and relative abundances based on different molecular methodologies. The upper panels show the correlations for picophytoplankton (size fraction 0.2–3 µm). The vertical axis corresponds to the relative biovolume based on flow cytometry (values displayed as % total biovolume of picophytoplankton), while the horizontal axis corresponds to relative read abundance based on molecular methods: 16S miTags (left upper panel) and *psbO* metagenomic counts (right upper panel). The lower panels show the correlations for nanophytoplankton (size fraction 5–20 μm). The vertical axis corresponds to the relative biovolume based on confocal microscopy quantification (values displayed as % total biovolume of eukaryotic phytoplankton), while the horizontal axis corresponds to relative read abundance based on molecular methods: V9‐18S rRNA gene metabarcoding (left middle panel), 18S rRNA gene miTags (left lower panel) and eukaryotic *psbO* metagenomic counts (right bottom panel). Spearman's correlation coefficients and *p*‐values are displayed in blue. Axis are in the same scale and the diagonal line corresponds to a 1:1 slope

We also compared the relative read abundances of the different marker genes against the proportional biovolumes for each taxon (Figure 4). Although the copy number of rRNA marker genes was previously proposed as a proxy of cell biovolume, the correlations of biovolume against rRNA gene relative abundances were not more consistent than those against *psbO* (Figure 4 and S12). The relative read abundances for *Prochlorococcus* and eukaryotic picophytoplankton based either on 16S rRNA or *psbO* were higher than their proportional biovolumes in the same samples, while the opposite was observed for *Synechococcus*. In the 5–20 µm size fraction, the biovolume proportion for haptophytes was clearly described by their *psbO* and 18S rRNA miTag relative abundances, while their V9‐18S rRNA gene reads were very low in relation to their biovolume. V9‐18S rRNA gene, 18S miTags and *psbO* reads were all correlated with relative biovolume for diatoms and dinoflagellates, but for the V9‐18S rRNA gene the data points were somewhat scattered and for 18S miTags and *psbO* the relative abundances for the reads were higher in relation to their biovolume. As the biovolume of other taxa was very low, their proportions of *psbO* and 18S miTags reads were much higher than the corresponding biovolume fraction, whereas there was no correlation between V9‐18S and biovolume.

### 3.5 Diversity analysis: Shannon‐index is robust to the biases introduced by the traditional molecular methods

**FIGURE 5:**
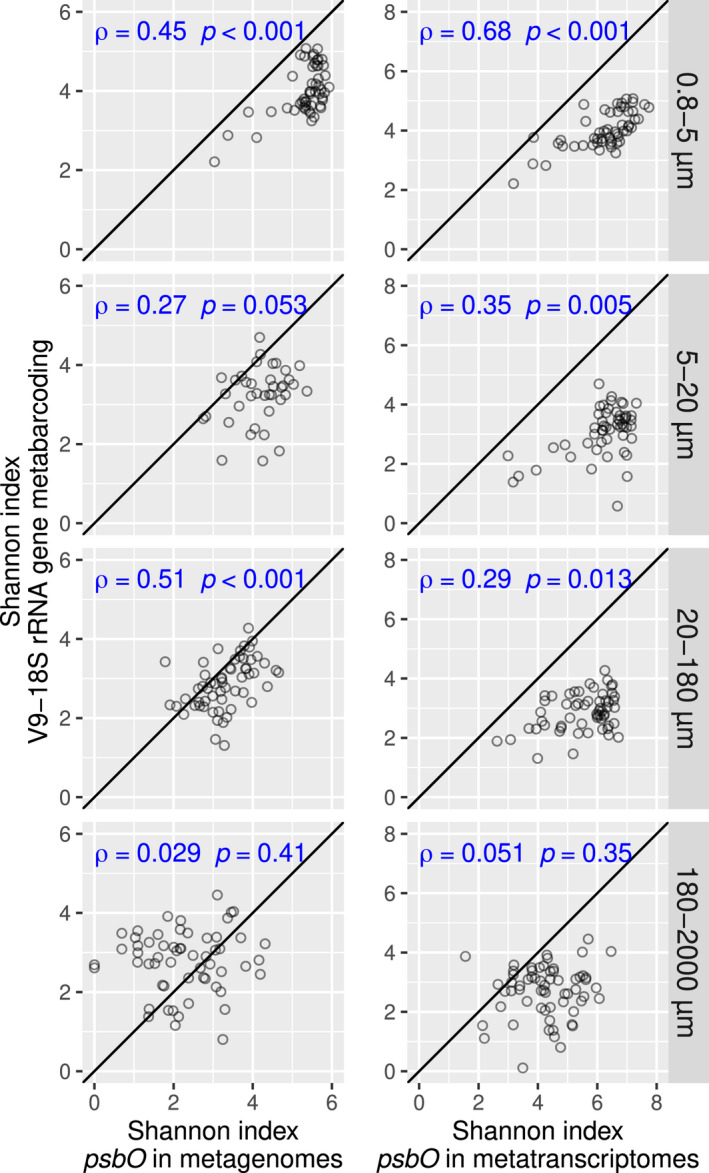
Correlation between the Shannon diversity values derived from different molecular methods for eukaryotic phytoplankton communities. The values derived from *psbO* metagenomics (left) and metatranscriptomics (right) were compared with those derived from V9–18S rRNA gene metabarcoding. Spearman's correlation coefficients and *p*‐values are displayed in blue. Axis are in the same scale and the diagonal line corresponds to a 1:1 slope

We further analysed whether our method improved the widely used Shannon index, a diversity index that accounts for both species richness and evenness (Calderón‐Sanou et al., 2020). We found a strong correlation between Shannon values for eukaryotic phytoplankton defined either by V9‐18S rRNA gene metabarcoding or by *psbO* metagenomics or metatranscriptomics (Figure 5). This is in agreement with previous reports showing no major effects of 16S rRNA gene copy number variation on the Shannon index of bacterial communities (Ibarbalz et al., 2019; Milanese et al., 2019). These results illustrate that not all subsequent analyses are affected by the biases introduced by traditional molecular methods.

### 3.6 Combining housekeeping and photosynthetic marker genes improves estimates of the distribution and abundance of phototrophs in a given taxonomic group

**FIGURE 6:**
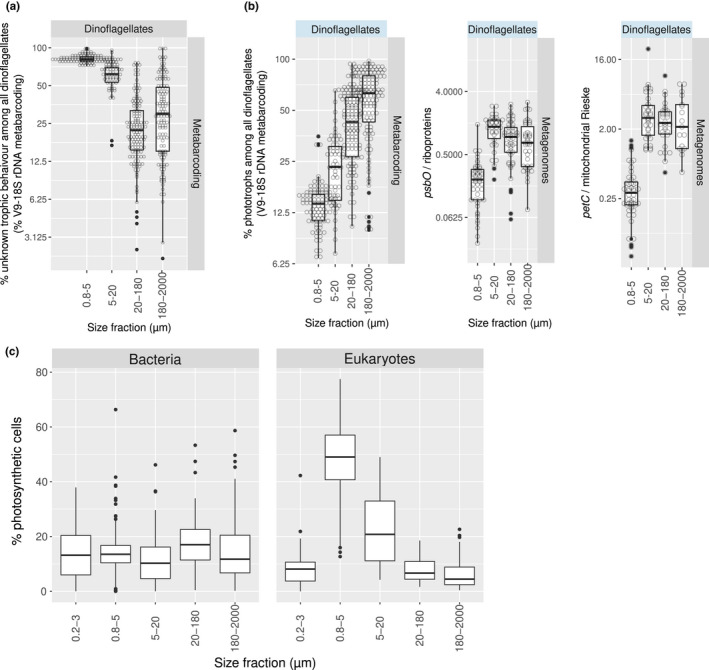
Variations in the abundance of phototrophs across size fractions. (a) Relative read abundance of V9–18S rRNA gene metabarcoding assigned to dinoflagellates of unknown capacity for photosynthesis. (b) Relative abundance of phototrophs among dinoflagellates based on different molecular methods. The first panel corresponds to the trait classification of V9–18S rRNA gene metabarcodes based on the literature (a description of the trait classification can be found at http://taraoceans.sb‐roscoff.fr/EukDiv/ and the trait reference database is available at https://zenodo.org/record/3768951#.YM4odnUzbuE). The second and third panels correspond to the ratio of metagenomic counts of photosynthetic *vs* housekeeping single‐copy nuclear‐encoded genes: *psbO* vs genes coding for ribosomal proteins, and the genes coding for the Rieske subunits of the Cyt bc‐type complexes from chloroplasts and mitochondria (i.e., *petC* and its mitochondrial homologue). (c) Relative abundance of phototrophs among bacterial and eukaryotic plankton across size fractions. The values were determined by the ratio of metagenomic counts of the single‐copy marker genes of photosynthesis (i.e., *psbO*) and housekeeping metabolism (i.e., *recA* for bacteria and genes encoding ribosomal proteins for eukaryotes)

To evaluate the uncertainties when inferring the photosynthesis trait using the taxonomy obtained from a nonphotosynthetic marker gene, we analysed the V9–18S OTUs assigned to dinoflagellates and found that most of their reads cannot be reliably classified as corresponding to a photosynthetic taxon or not (Figure 6a), especially for those OTUs whose taxonomic affiliation is “unknown dinoflagellate” (Figure S13). The uncertainty was especially significant in the 0.8–5 µm size fraction, where on average ~80% of the total dinoflagellate read abundance remained unclassified (Figure 5a and S13).

Therefore, as well as finding a more relevant marker gene for phytoplankton, we also propose combining it with established single‐copy housekeeping genes (specifically *recA* for bacteria (Sunagawa et al., 2013) and genes encoding ribosomal proteins for eukaryotes (Carradec et al., 2018; Ciccarelli et al., 2006)), to estimate the fraction of photosynthetic members in a given community or within a specific clade. In the case of eukaryotes, a set of genes of interest for this aim are *petC* and its mitochondrial homologues (i.e., the nuclear‐encoded genes for the Rieske subunits of the Cyt *bc*‐type complexes from chloroplasts and mitochondria) (Table 2 and Figure S5). As an example, we analysed the distribution of phototrophy across size fractions among the eukaryotic groups under study. As expected, it did not reveal any differences for diatoms, haptophytes, chlorophytes or pelagophytes (Figure S14), reflecting the relative paucity of described secondarily nonphotosynthetic members of these groups. However, for dinoflagellates we observed a significant proportion of non‐photosynthetic lineages in the 0.8–5 µm size‐fraction in comparison with the other size ranges, which were also shown by the V9‐18S rRNA gene metabarcoding method (Figure 5b, S13 and S14). However, whereas the metabarcoding data showed a dramatic increase in phototrophs towards the larger size classes of dinoflagellates, the metagenomic analysis showed similar levels between the three larger size fractions (5–20 µm, 20–180 µm, 180–2,000 µm) (Figure 6b). These different patterns between the two marker genes might be explained by differences in the unknown trait assignment of the 18S rRNA gene barcodes and/or in the 18S rRNA gene copy number (e.g., higher copy numbers in photosynthetic species in larger size fractions).

The approach suggested can be applied to unveil variation of phototrophs in whole plankton communities, including both bacteria and eukaryotes. In order to do so, we mapped the metagenomic reads against our comprehensive catalogue of *psbO* sequences (Figure S1). The highest proportion of phytoplankton among eukaryotes was observed in the 0.8–5 µm size fraction, followed by the 5–20 µm size‐fraction, while the lowest value was found in the 180–2,000 µm size range (Figure 6c), where copepods are prevalent (considered one of the most abundant animals on the planet). Surprisingly, the percentage of phototrophs among bacterioplankton did not vary across size fractions (10%–15% on average; see next section). In the 0.2–3 µm size fraction, very similar values were detected by 16S miTags, but when comparing both molecular methods with flow cytometry, the *psbO*/*recA* ratio was better correlated to flow cytometry (Spearman's Rho of 0.82 vs. 0.91, *p* <.001, and a closer 1:1 relationship) (Figure S15).

### 3.7 Trans‐domain comparison reveals unexpected abundance of picocyanobacteria in large size fractions

**FIGURE 7:**
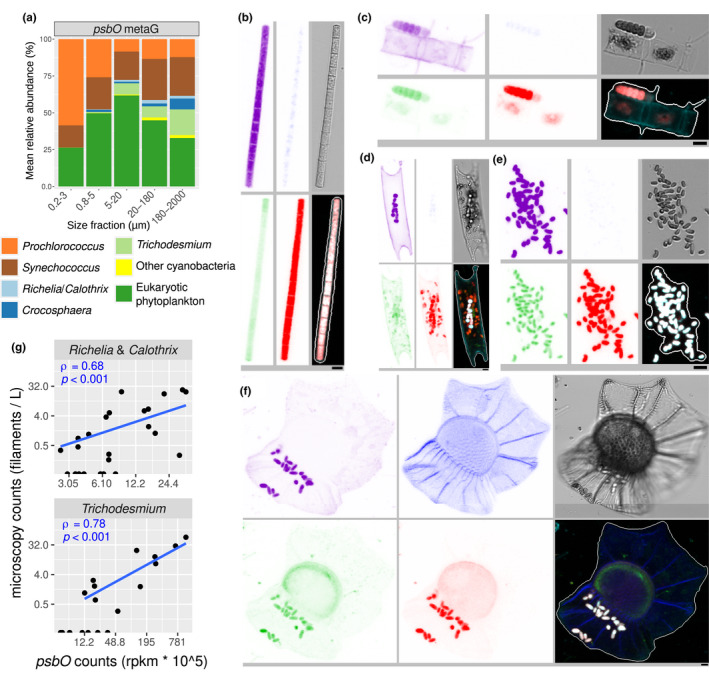
Prokaryotic and eukaryotic phytoplankton community structure across the entire plankton size spectrum. (a) Average relative cell abundance of phototrophs across all metagenomes based on *psbO* metagenomic reads. (b–f) Examples of confocal microscopy detection of cyanobacteria in the 20–180 µm size fraction. From top left to bottom right, the displayed channels for each micrograph correspond to cell surface (cyan, AlexaFluor 546 dye), DNA (and the theca in dinoflagellates) (blue, Hoechst dye), cellular membranes (green, DiOC6 dye), chlorophyll autofluorescence (red), bright field, and all merged channels. The size bar at the bottom left of each microscopy image corresponds to 2.5 μm. (b) *Trichodesmium* filament. (c) *Calothrix* filament outside a chain of the diatom *Chaetoceros* sp. (d) *Richelia* filaments inside the diatom *Eucampia cornuta*. (e) Picocyanobacterial aggregate. (f) Picocyanobacterial symbionts in the dinoflagellate *Ornithocercus thumii*. (g) Correlation analysis between *Trichodesmium* and *Richelia*/*Calothrix* quantifications by confocal microscopy and *psbO* metagenomic reads in size fraction 20–180 µm. Spearman Rho's correlations coefficients and *p*‐values are indicated. rpkm: reads per kilobase per million mapped reads

To further examine the distribution of both prokaryotic and eukaryotic phytoplankton across the whole size spectrum, we continued the analysis of the mapped metagenomic reads against our catalogue of *psbO* sequences (Figure S1). We observed a high abundance of cyanobacteria in the large size fractions in relation to the eukaryotic phytoplankton (Figure 7a). The nitrogen‐fixers *Trichodesmium* and *Richelia*/*Calothrix* were found principally in the 20–180 and 180–2,000 μm size fractions (Figure 6a), which is expected as the former forms filaments and colonies while the second group are symbionts of certain diatoms (Figure 7b–d). These genera were recently quantified in the high‐throughput confocal microscopy data set from the 20–80 µm size fraction (Pierella Karlusich et al., 2021). We therefore checked the correlations of these data with the *psbO* determinations and found them to be very strongly related (Figure 7g).

**FIGURE 8:**
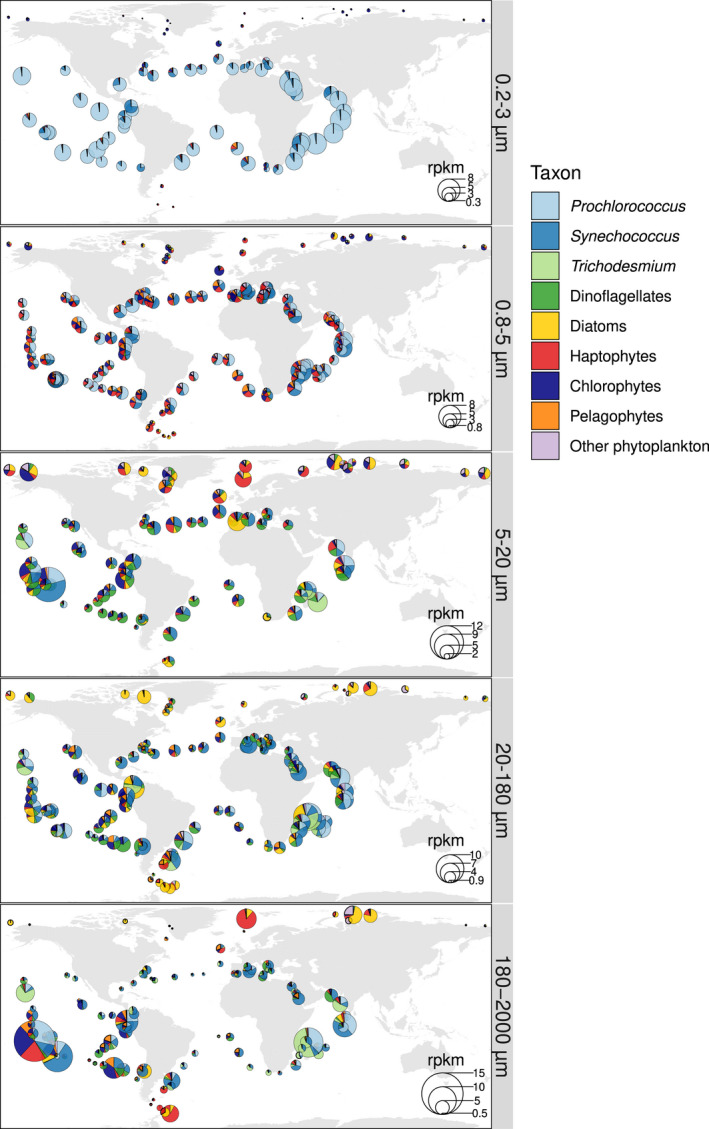
Global biogeographical patterns of marine phytoplankton in surface waters. The pie charts show the *psbO* relative abundance of the main cyanobacteria and eukaryotic phytoplankton in metagenomes derived from different size‐fractionated samples. Values are displayed as rpkm (reads per kilobase per million mapped reads). The comparison between the *psbO*‐based relative cell abundances versus the patterns corrected by biovolume are displayed in Figure S16. The distribution of the main phytoplankton groups in the size fraction in which they were most prevalent is shown in Figure S17

To our surprise, we also detected a high abundance of both *Prochlorococcus* and, in particular, *Synechococcus*, in the large size fractions (Figure 7a) across multiple and geographically distinct basins of the tropical and subtropical regions of the world's ocean (Figure 8). Picocyanobacteria have small cell diameters (<1 μm), and therefore should readily pass through the filters with pore sizes of 5, 20 or 180 μm. Although smaller cells can get caught on larger filters, their abundance should be limited and hence not responsible for the values observed. The reason why a substantial fraction of picocyanobacteria were found in the largest size fractions may be colony formation, symbiosis, attachment to particles, or their grazing by protists, copepods and/or suspension feeders. We examined these possibilities by looking at the *Tara* Oceans confocal microscopy data set, and found many microscopy images evidencing colony formation and symbiosis in the 20–80 μm size fraction (Figure 7e–f). This is in agreement with the mapping of the *Tara* Oceans metagenomes against a recently sequenced single cell genome of a *Synechococcus* living as a dinoflagellate symbiont (Nakayama et al., 2019). In addition, there are reports of picocyanobacterial symbionts among isolates of planktonic foraminifers, radiolarians, tintinnids, and dinoflagellates (Bird et al., 2017; Foster et al., 2006; Kim et al., 2021; Yuasa et al., 2012) and picocyanobacterial colonies were observed in a regional study based on optical methods (Masquelier & Vaulot, 2008) and in laboratory cultures (Deng et al., 2015, 2016).

These results suggest that we should move from the traditional view of *Synechococcus*/*Prochlorococcus* as being exclusively part of picoplankton communities, and instead should consider them as part of a broader range of the plankton size spectrum (in a similar way as occurs with other small‐celled phytoplankton such as the haptophyte *Phaeocystis* [Beardall et al., 2009; Decelle et al., 2019]). However, it should be borne in mind that these results correspond to estimates of relative cell abundance, and thus the picture is very different when translated to biovolume or biomass, due to the large differences in cell size (Figure S16). All in all, our approach allows us to make trans‐domain comparisons, which can reveal photosymbiosis and cell aggregates (Figure 7), and allows us to examine the biogeography of the entire phytoplankton community simultaneously (Figure 8, S16 and S17).

## 4 DISCUSSION

We searched for core photosynthetic, single‐copy, nuclear genes in genomes and transcriptomes of cultured phytoplankton strains for their use as marker genes. Of five resulting candidates, *psbO* emerged as the most suitable due to its lack of nonphotosynthetic homologues (but note that the other genes could be incorporated in future studies by discarding nonphotosynthetic homologues by phylogenetic and/or sequence similarity methods). We applied this new approach by retrieving *psbO* sequences from the metagenomes generated by *Tara* Oceans, and successfully validated it using the optical determinations from the same expedition.

We also compared the *psbO* patterns with those of rRNA genes, which are the most widely used taxonomic markers for plankton due to many advantages: universality and phylogenetic informativeness at different taxonomic levels, high representation in reference databases, ease of amplification due to their abundance (e.g., multiple copies), etc. When compared with V9‐18S metabarcoding data, our approach yields lower abundances for diatoms and dinoflagellates at the expense of higher abundances of haptophytes, chlorophytes and pelagophytes. These results were remarkably consistent with those obtained by microscopy. To disentangle the effect of PCR bias versus copy number in the patterns of V9‐18S metabarcoding, we generated 18S miTags from the analysed metagenomes and obtained improved correlations more similar to those based on *psbO*, suggesting an strong effect of PCR bias. It is also important to take into account that not all analyses are affected by the biases introduced by traditional molecular methods, as we showed for the Shannon index. In relation to the 16S rRNA gene, the current study only analysed the sequences retrieved from metagenomes (e.g., 16S miTags) generated from the 0.2–3 µm size fraction, where most picoplankton cells only have a single chloroplast. This probably explains the good correlations between 16S and flow cytometry, which were anyhow stronger with *psbO*. In the future it will be of interest to analyse the relative abundances of 16S rRNA genes in the larger size fractions, which usually contains problematic taxa for this marker gene, including those that introduce high copy number biases due to the presence of multiple plastids (e.g., centric diatoms) or those that are not detected due to their divergent 16S genes (e.g., dinoflagellates and chrompodellids).

While our study demonstrated that *psbO* reflects the relative cell abundance of phytoplankton, some previous studies suggested that rRNA genes reflect the relative biovolume of the corresponding taxa. However, there is still no clear consensus for rRNA genes as proxies of biovolume. Here, we observed that the biovolume proportions for diatoms, dinoflagellates and haptophytes were described by their *psbO* or 18S miTags relative abundance, while the biovolume proportions of other taxa were not captured clearly by either marker gene. In addition, all the patterns for V9‐18S metabarcoding were weak in comparison with *psbO* or 18S miTags. Among picoplankton, interpreting the relative read abundances of *psbO* or 16S as proxies of biovolume would result in overestimations of *Prochlorococcus* and photosynthetic eukaryotes at the expense of *Synechococcus*.

In addition to our methodological insights, we revealed unexpected ecological features of marine phytoplankton. For example, our trans‐domain comparison detected picocyanobacteria in high numbers in large size fractions, which was supported by the observation of numerous images of picocyanobacterial aggregates and endosymbionts in the *Tara* Oceans imaging data set. Moreover, we analysed the abundance of *psbO* in relation to the average abundance of single‐copy housekeeping genes to quantify the relative contribution of phototrophs in a given taxon, observing that small dinoflagellates (0.8–5 µm) are mainly heterotrophic, while those in the larger size communities (>5 µm) are mainly photosynthetic. All these patterns from size‐fractionated samples can be complemented in the future by exploring non‐fractionated metagenomes, such as those from BioGeotraces (Biller et al., 2018).

In addition to metagenomes, we also analysed *psbO* in metatranscriptomes, where dinoflagellates stood out from the rest due to their much higher *psbO* abundance ratio of mRNA abundance to gene copy number. It will be of interest to analyse if this reflects higher “photosynthetic activity” or if it is an effect of their predominant post‐transcriptional regulation (Cohen et al., 2021; Roy et al., 2018). In addition, the analyses of metatranscriptomes can give clues about mixotrophy. For example, it will be of interest to detect changes in the abundance ratio of *psbO* to housekeeping genes between metatranscriptomes and metagenomes for use as an index of mixotrophy.

The very deep sequencing of the *Tara* Oceans metagenomes (between ~10^8^ and ~10^9^ total reads per sample) allowed us to carry out taxonomic analysis based on a unique gene, in spite of dilution of the signal. As reduced DNA sequencing costs are leading to the replacement of amplicon‐based methods by metagenome sequencing, we expect the utility of our method to increase in future years. In the short term, a barcode approach using *psbO* primers is a promising cheap alternative, although it will be subject to PCR biases and affected by the presence of introns.

It is important to note that *psbO* can be used to estimate absolute cell abundances with careful normalization and quantitative DNA extraction methods. In the current study we did not attempt to do it because the metagenomic sampling from *Tara* Oceans was not specifically designed to quantify metagenomic signals per seawater volume due to the lack of “spike‐ins” (e.g., DNA internal standards).

The use of functional genes as taxonomic markers for phytoplankton has been restricted to some surveys (using plastid‐encoded genes) (Farrant et al., 2016; Man‐Aharonovich et al., 2010; Paul et al., 2000; Zeidner et al., 2003). This is not the case for other functional groups, such as nitrogen‐fixers, which are studied by targeting a gene encoding a subunit of the nitrogenase enzymatic complex (Zehr & Paerl, 1998) and for which extensive reference sequence databases are now available (https://www.jzehrlab.com; Heller et al., 2014). To facilitate the incorporation of *psbO* into future molecular‐based surveys, we have generated a database of >18,000 annotated *psbO* sequences (https://www.ebi.ac.uk/biostudies/studies/S‐BSST659; Figure S1). We hope that the release of this data, and the establishment of *psbO* as a new biomarker for quantifying species abundances, will open new perspectives for molecular‐based evaluations of phytoplankton communities.

Based on the current analyses, we recommend the use of *psbO* as a proxy of relative cell abundance of the whole phytoplankton community. However, analyses such as Shannon index are robust enough to be based on rRNA genes. Finally, we did not find a good proxy of relative phytoplankton biovolume among the analysed molecular approaches (*psbO*, V9–18S metabarcoding, 18S and 16S miTags), indicating that optical methods are still the recommended method for biovolume.

## AUTHOR CONTRIBUTIONS

Juan José Pierella Karlusich and Chris Bowler designed the project. Juan José Pierella Karlusich conducted the study and performed the primary data analysis and visualization. Juan José Pierella Karlusich compiled the *psbO* gene reference catalogue and Eric Pelletier performed the metagenomic mapping on it. Eric Pelletier and Nicolas Henry generated the 18S miTags data set. Richard G. Dorrell carried out the phylogenetic‐based annotation of 16S rRNA gene OTUs. Fabien Lombard, Sébastien Colin and Colomban de Vargas assisted with the confocal microscopy data set, Adriana Zingone and Eleonora Scalco with the optical microscopy and Josep M. Gasol and Silvia G. Acinas with the flow cytometry. All authors assisted with interpretation of the data. Juan José Pierella Karlusich and Chris Bowler wrote the manuscript with substantial input from all authors.

## BENEFIT‐SHARING STATEMENT

Benefits from this research accrue from the sharing of our data and results on public databases as described above.

## Supporting information

Supplementary Material

## Data Availability

All datasets analysed for this study are of public access as described in Table 1. The curated *psbO* database was submitted to the EMBL‐EBI repository BioStudies (www.ebi.ac.uk/biostudies) under accession S‐BSST659. The 18S miTags data set covering size fractions between 0.8 and 2,000 µm was submitted to the same repository under accession S‐BSST762. The Supporting Information tables for flow cytometry and optical microscopy as well as the protein sequences used for building the protein similarity networks are available at S‐BSST761.
