# Supplementary material for: A robust approach to estimate relative phytoplankton cell abundances from metagenomes
Source: Mol Ecol Resour. 2022 Feb 16;23(1):16–40. doi: 10.1111/1755-0998.13592 (PMC10078663; doi:10.1111/1755-0998.13592)
Supplement: Supplementary file 1 — Supplementary Material [file MEN-23-16-s001.pdf]

MOLECULAR ECOLOGY RESOURCES

Supplemental Information for:

A robust approach to estimate relative phytoplankton cell abundances from metagenomes

Juan José Pierella Karlusich[1,2 *], Eric Pelletier[2,3], Lucie Zinger[1], Fabien Lombard[2,4,5], Adriana Zingone[6], Sébastien Colin[7,8,9], Josep M. Gasol[10], Richard G. Dorrell[1], Nicolas Henry[2,11], Eleonora Scalco[6], Silvia G. Acinas[10], Patrick Wincker[2,3], Colomban de Vargas[2,8], Chris Bowler[1,2 *]

[1] Institut de Biologie de l'ENS (IBENS), Département de biologie, École normale supérieure, CNRS, INSERM, Université PSL, 75005 Paris, France
[2] CNRS Research Federation for the study of Global Ocean Systems Ecology and Evolution, FR2022/Tara Oceans GOSEE, 3 rue Michel-Ange, 75016 Paris, France
[3] Génomique Métabolique, Genoscope, Institut François Jacob, CEA, CNRS, Univ Evry, Université Paris-Saclay, 91057 Evry, France
[4] Sorbonne Universités, CNRS, Laboratoire d'Océanographie de Villefranche (LOV), 06230 Villefranche-sur-Mer, France
[5] Institut Universitaire de France (IUF), Paris, France
[6] Stazione Zoologica Anton Dohrn, Villa Comunale, 80121 Naples, Italy
[7] European Molecular Biology Laboratory, Heidelberg, Germany
[8] Sorbonne Université, CNRS, Station Biologique de Roscoff, UMR 7144, ECOMAP, 29680 Roscoff, France
[9] Max Planck Institute for Developmental Biology, Tübingen, Germany
[10] Department of Marine Biology and Oceanography, Institut de Ciènces del Mar, CSIC, Barcelona, Spain
[11] CNRS, Sorbonne Université, FR2424, ABiMS, Station Biologique de Roscoff, 29680 Roscoff, France

***corresponding authors:**
Juan José Pierella Karlusich (pierella@biologie.ens.fr)
and Chris Bowler (cbowler@biologie.ens.fr)

MOLECULAR ECOLOGY RESOURCES

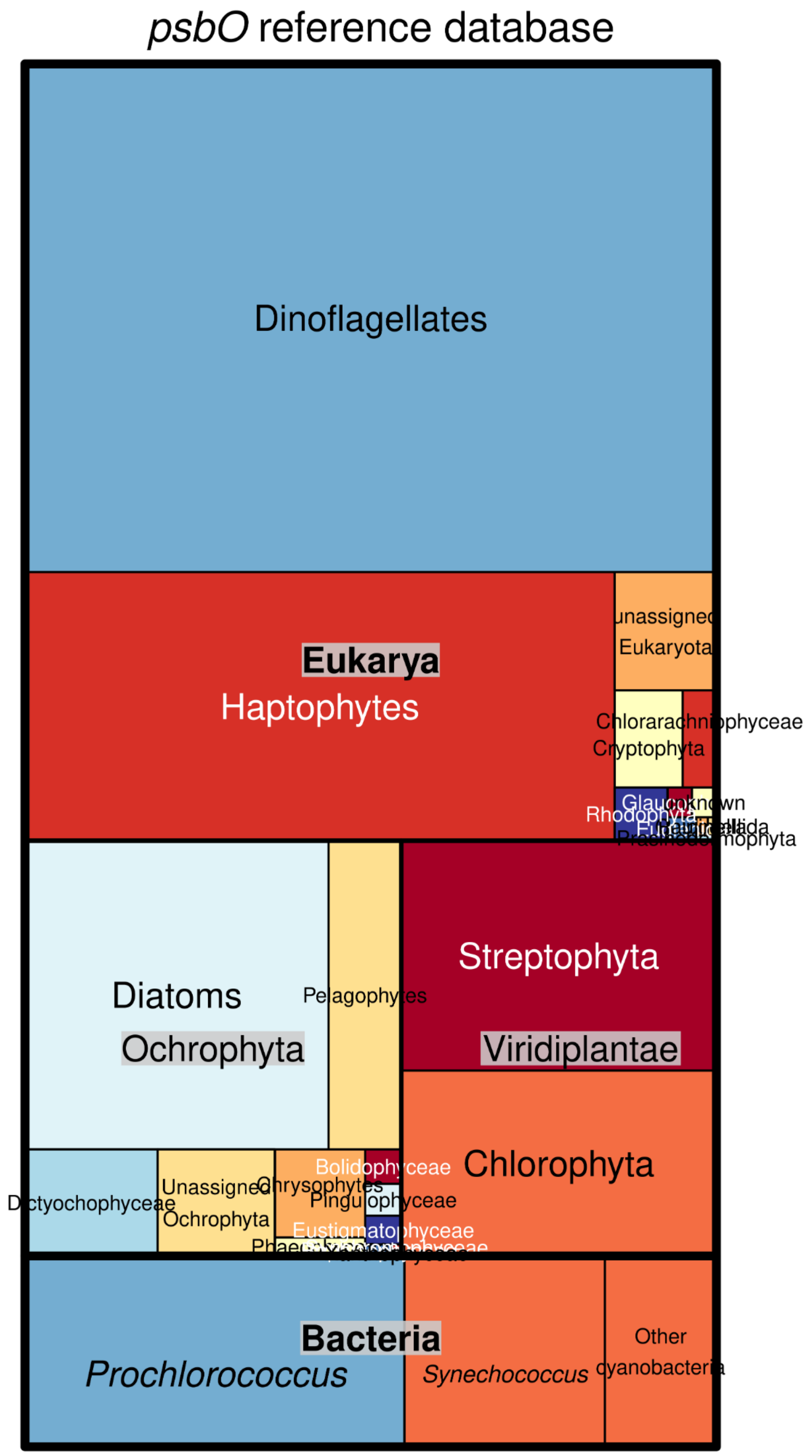

Figure S1: Taxonomic distribution of the curated *psbO* database generated in the current work. It consists of 18,378 unique sequences covering cyanobacteria, photosynthetic protists, macroalgae and land plants. The sequences were retrieved from sequenced genomes and transcriptomes from cultured isolates as well as from the environmental sequence catalogs of Global Ocean Sampling (Rusch et al. 2007) and *Tara* Oceans (Salazar et al. 2019; Carradec et al. 2018; Delmont et al. 2020; 2021). The database can be downloaded from the EMBL-EBI repository BioStudies (www.ebi.ac.uk/biostudies) under accession S-BSST659.

MOLECULAR ECOLOGY RESOURCES

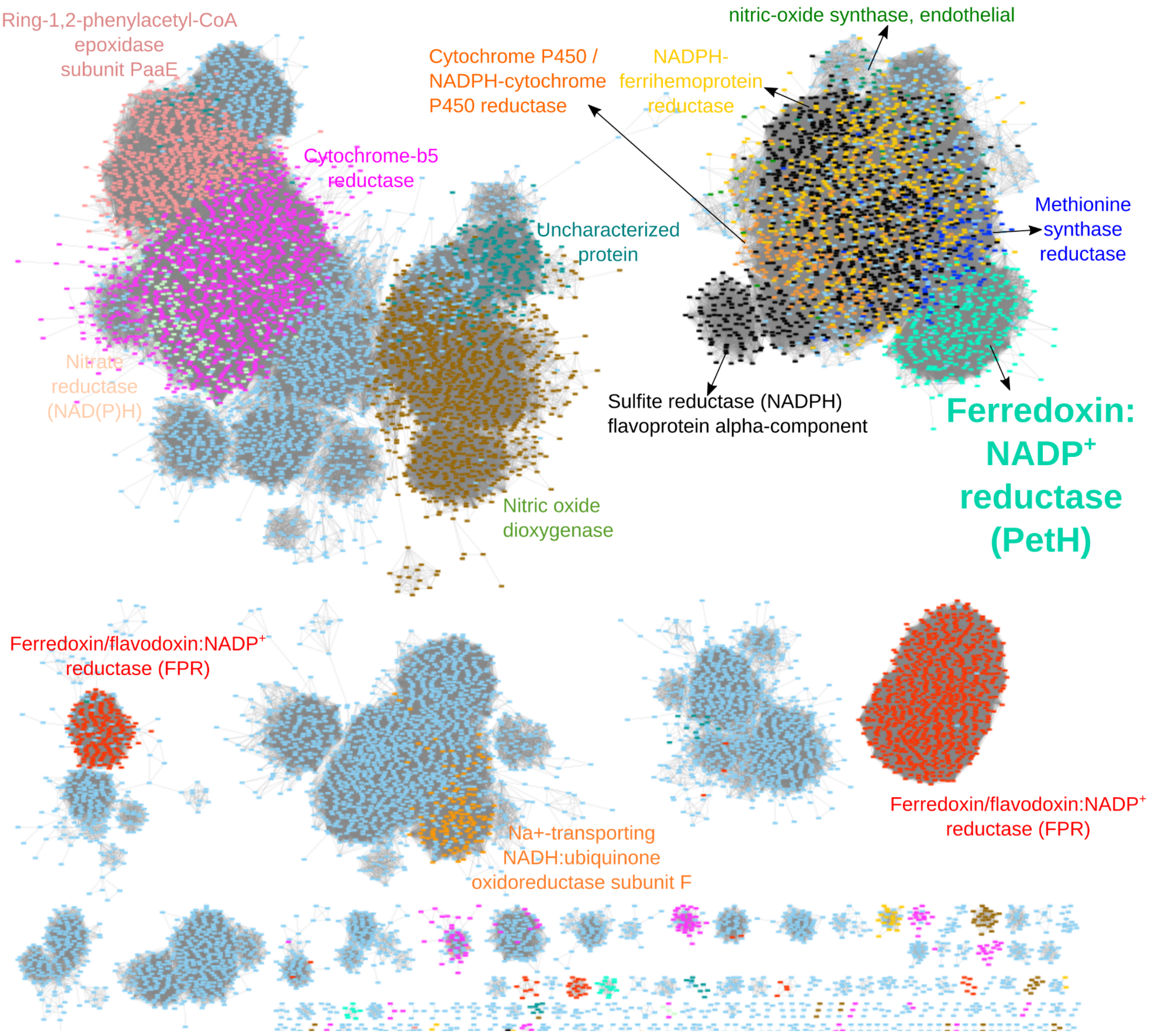

Figure S2: Sequence analysis of ferredoxin:NADP$^+$ reductase (PetH) and homologs using a protein similarity network for the Pfam domain NAD_binding_1 (PF00175). Each node corresponds to a representative sequence (clustered at 80% identity by CDHIT) and those sequences with similarity higher than a score cutoff are linked (score cut-off of 22 in blastp alignment). The network was built with sequences retrieved from the literature and from reference genomes and transcriptomes. Nodes are coloured according to their functional assignment based on BlastKOALA. The nodes for FNR are in light green, and includes photosynthetic FNRs as well as FNRs involved in nitrogen metabolism and in non-photosynthetic plastids (Pierella Karlusich & Carrillo 2017 *Phot Res* 134:235–250) and FNR from heterotrophic bacteria acquired by horizontal gene transfer (Catalano Dupuy et al. 2011 *PLoS One* 6:e26736)..

MOLECULAR ECOLOGY
RESOURCES

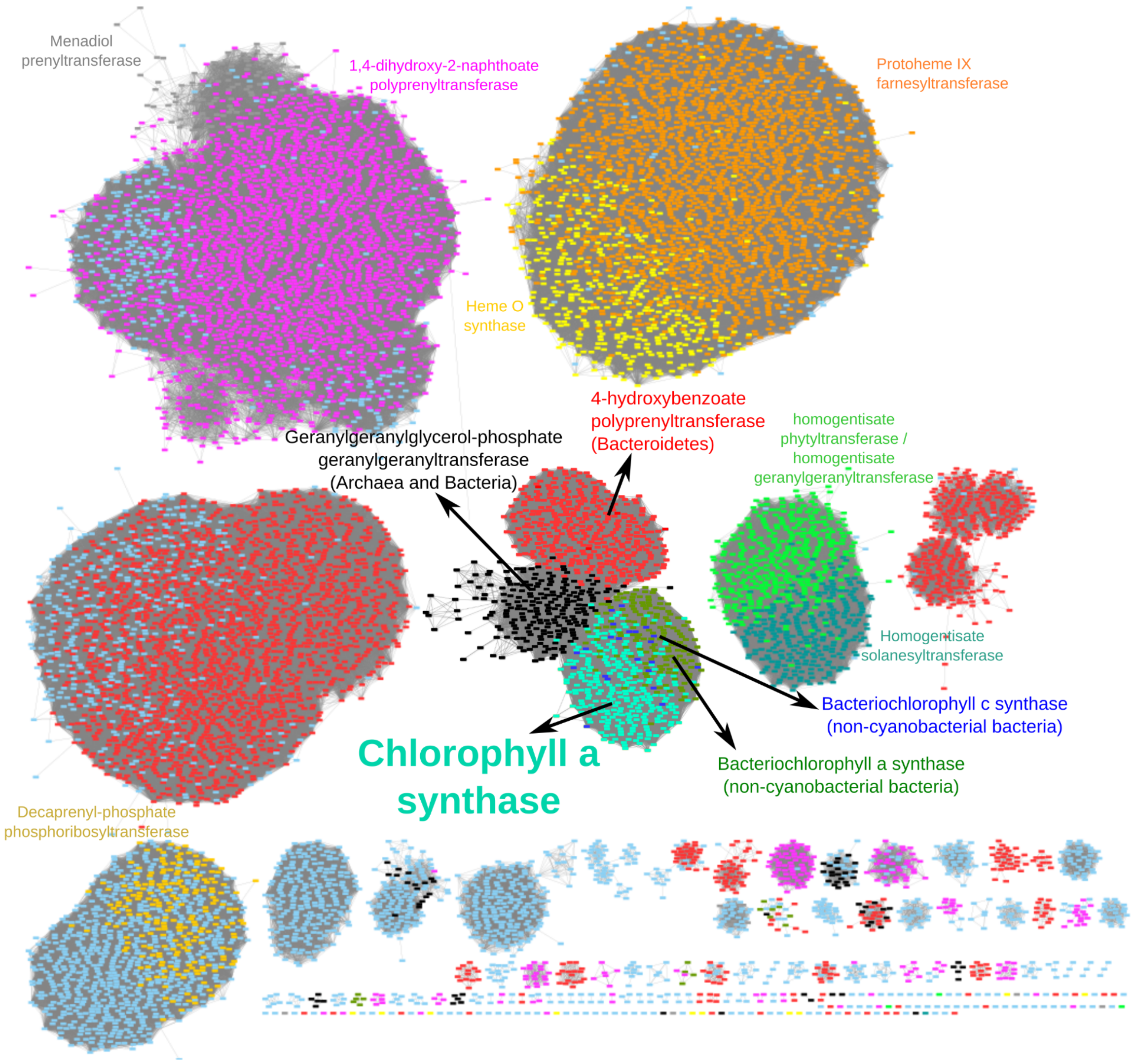

Figure S3: Sequence analysis of chlorophyll *a* synthase (ChlG) and homologs using a protein similarity network for the Pfam domain UbiA (PF01040). Each node corresponds to a representative sequence (clustered at 80% identity by CDHIT) and those sequences with similarity higher than a score cutoff are linked (score cut-off of 25 in blastp alignment). The network was built with sequences retrieved from the literature and from reference genomes and transcriptomes. Nodes are coloured according to their functional assignment based on BlastKOALA.

MOLECULAR ECOLOGY
RESOURCES

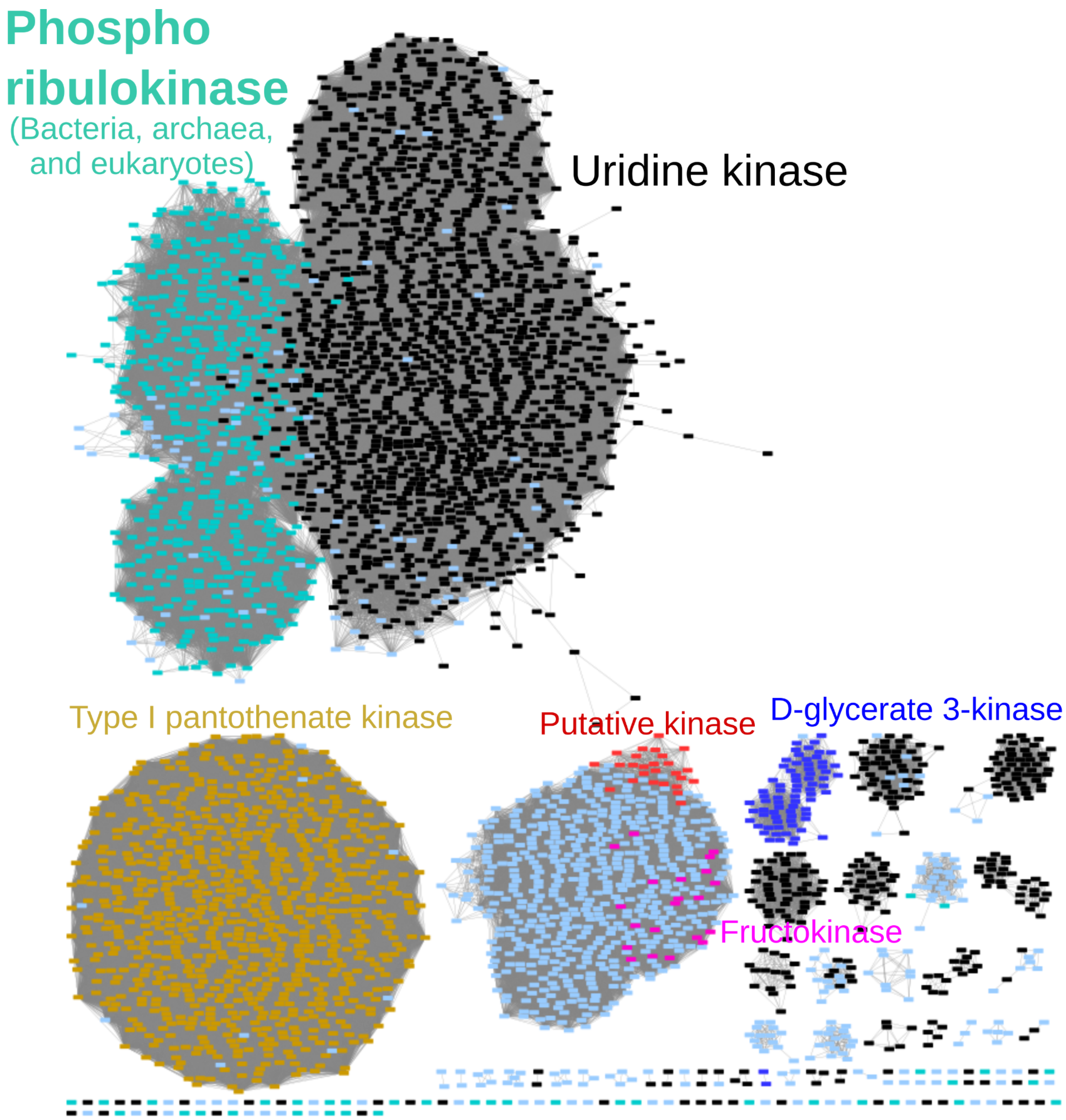

Figure S4: Sequence analysis of phosphoribulokinase (PRK) and homologs using a protein similarity network for the Pfam domain PRK (PF00485). Each node corresponds to a representative sequence (clustered at 80% identity by CDHIT) and those sequences with similarity higher than a score cutoff are linked (score cut-off of 25 in blastp alignment). The network was built with sequences retrieved from the literature and from reference genomes and transcriptomes. Nodes are coloured according to their functional assignment based on BlastKOALA. The nodes for PRK are in light green, and include photosynthetic PRKs as well as those from archaea and non-cyanobacterial bacteria.

MOLECULAR ECOLOGY RESOURCES

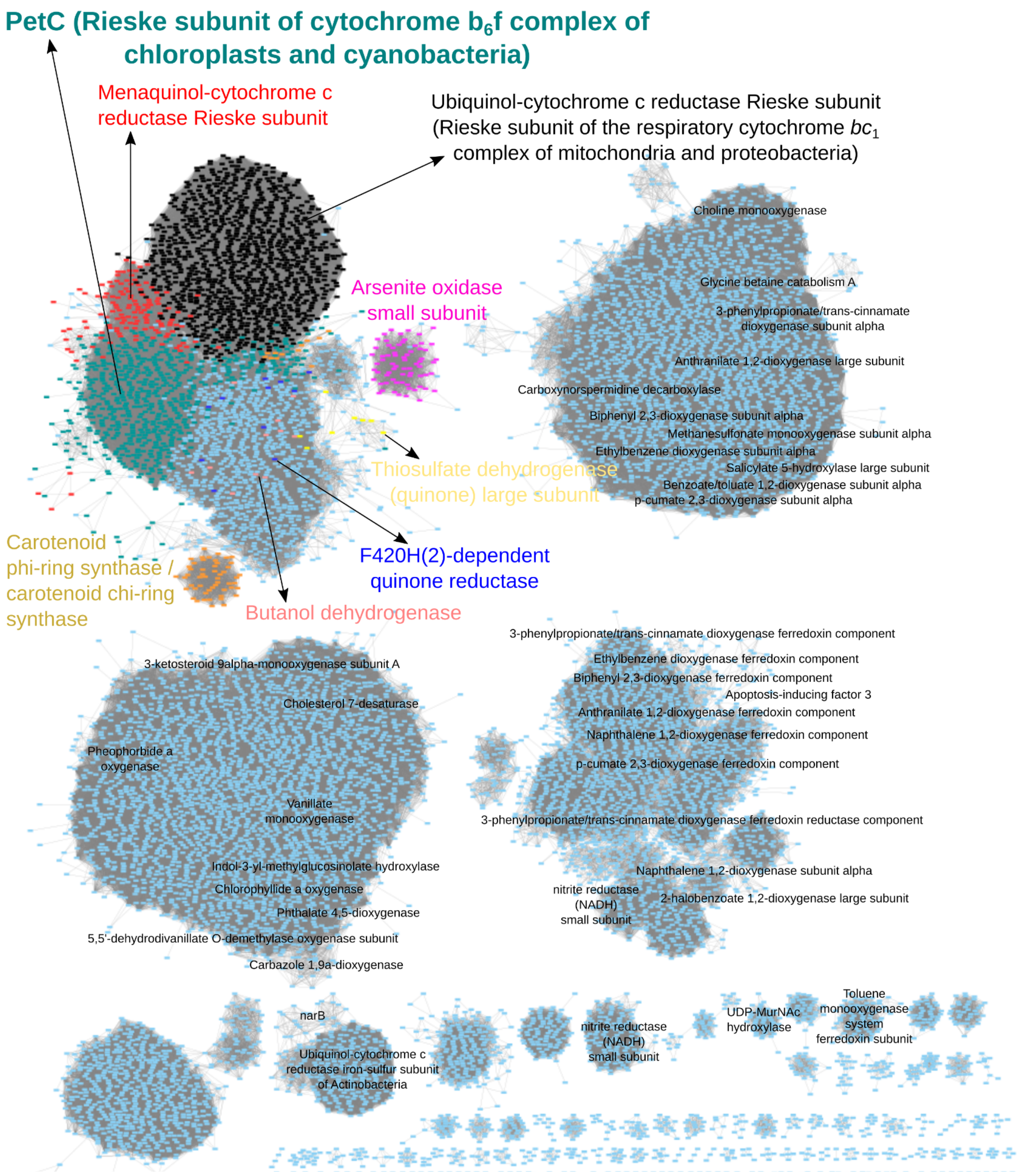

Figure S5: Sequence analysis of PetC (Rieske subunit of the Cytochrome b_6f complex) and homologs using a protein similarity network for the Pfam domain Rieske (PF00355). Each node corresponds to a representative sequence (clustered at 80% identity by CDHIT) and those sequences with similarity higher than a score cutoff are linked (score cut-off of 18 in blastp alignment). The network was built with sequences retrieved from the literature and from reference genomes and transcriptomes. Labels correspond to the functional assignment based on BlastKOALA, and for the cluster of interest nodes are coloured according to the functional assignment of their sequences. The nodes for PetC are in green and those for the Rieske subunit of the respiratory Cytochrome bc_1 complex are in black.

MOLECULAR ECOLOGY
RESOURCES

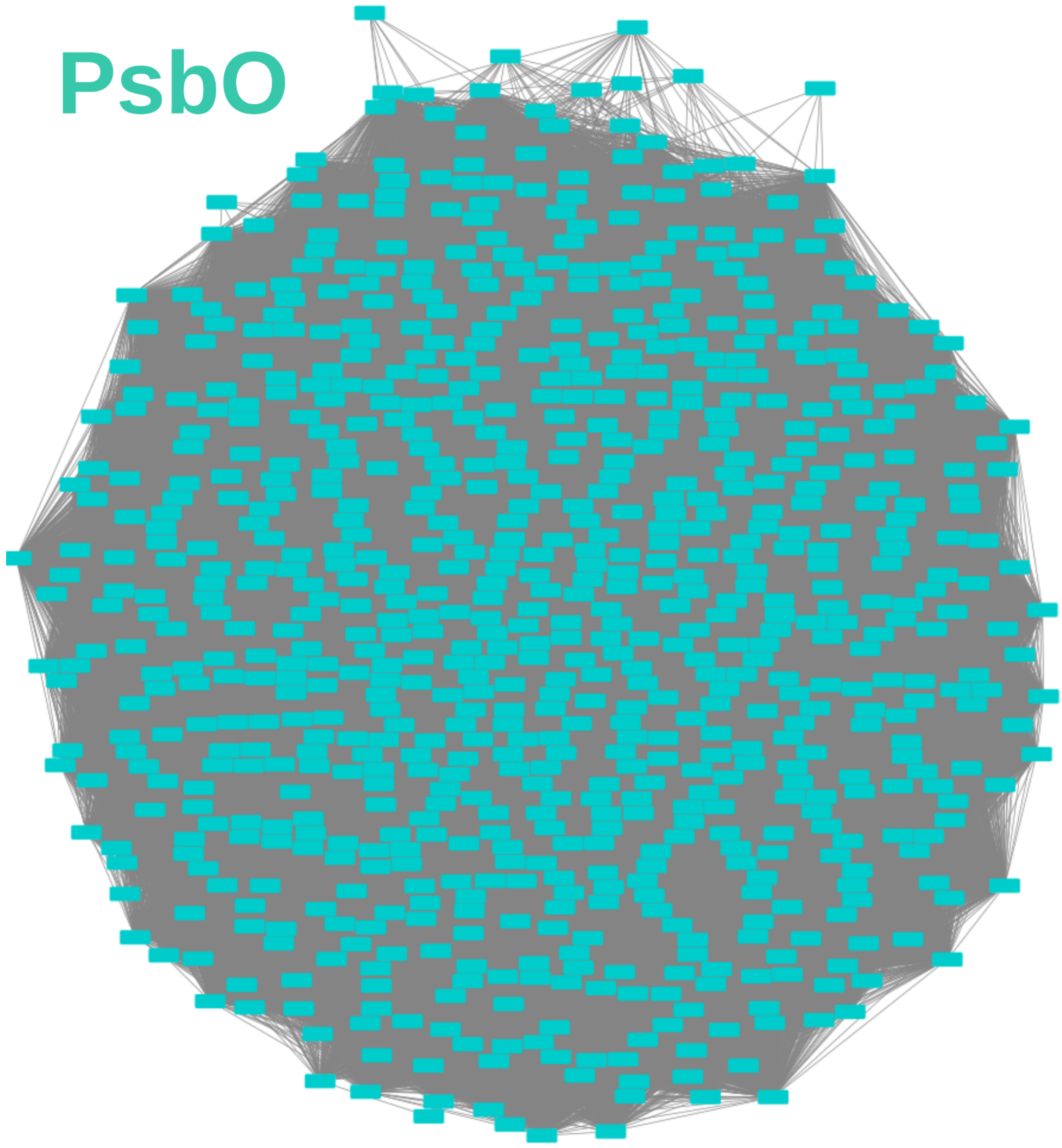

Figure S6: Sequence analysis of PsbO protein using a protein similarity network for the Pfam domain MSP (PF01716). Each node corresponds to a representative sequence (clustered at 80% identity by CDHIT) and those sequences with similarity higher than a score cutoff are linked (score cut-off of 30 in blastp alignment). The network was built with sequences retrieved from the literature and from reference genomes and transcriptomes. Nodes are coloured according to their functional assignment based on BlastKOALA.

MOLECULAR ECOLOGY RESOURCES

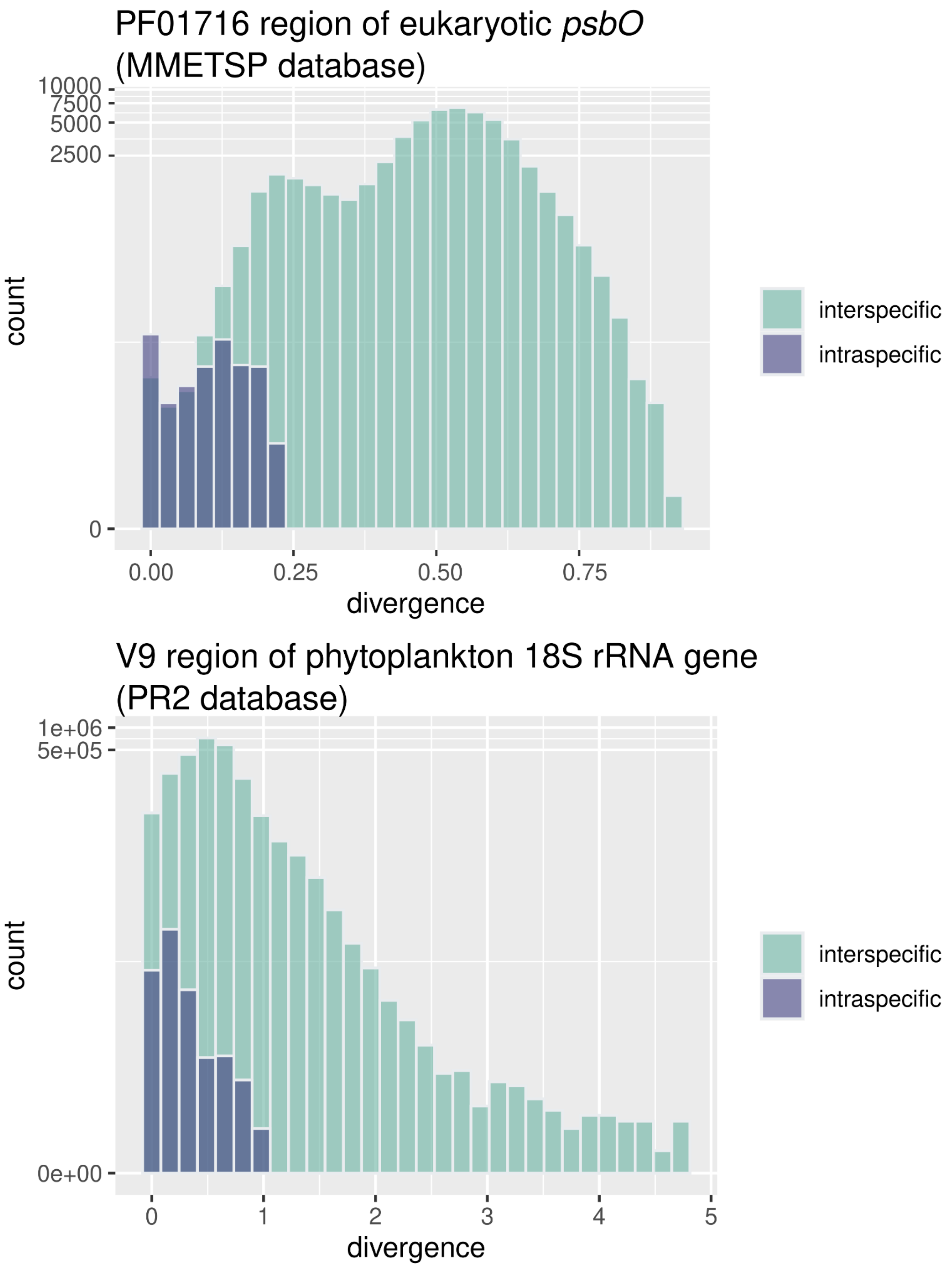

Figure S7: Intraspecific (blue) and interspecific (green) variation in genetic distances of eukaryotic phytoplankton sequences for the region of *psbO* coding for the Pfam domain PF01716 (upper panel) and the V9 region of 18S rRNA gene (lower panel). Sequences were retrieved from MMETSP project (Keeling et al. 2014) for *psbO* and from PR2 database (Guillou et al. 2012) for V9-18S maker. In this later case, the sequence assigned to phytoplankton were selected based on a public functional database available at https://zenodo.org/record/3768951#.YM4odnUzbuE.

MOLECULAR ECOLOGY
RESOURCES

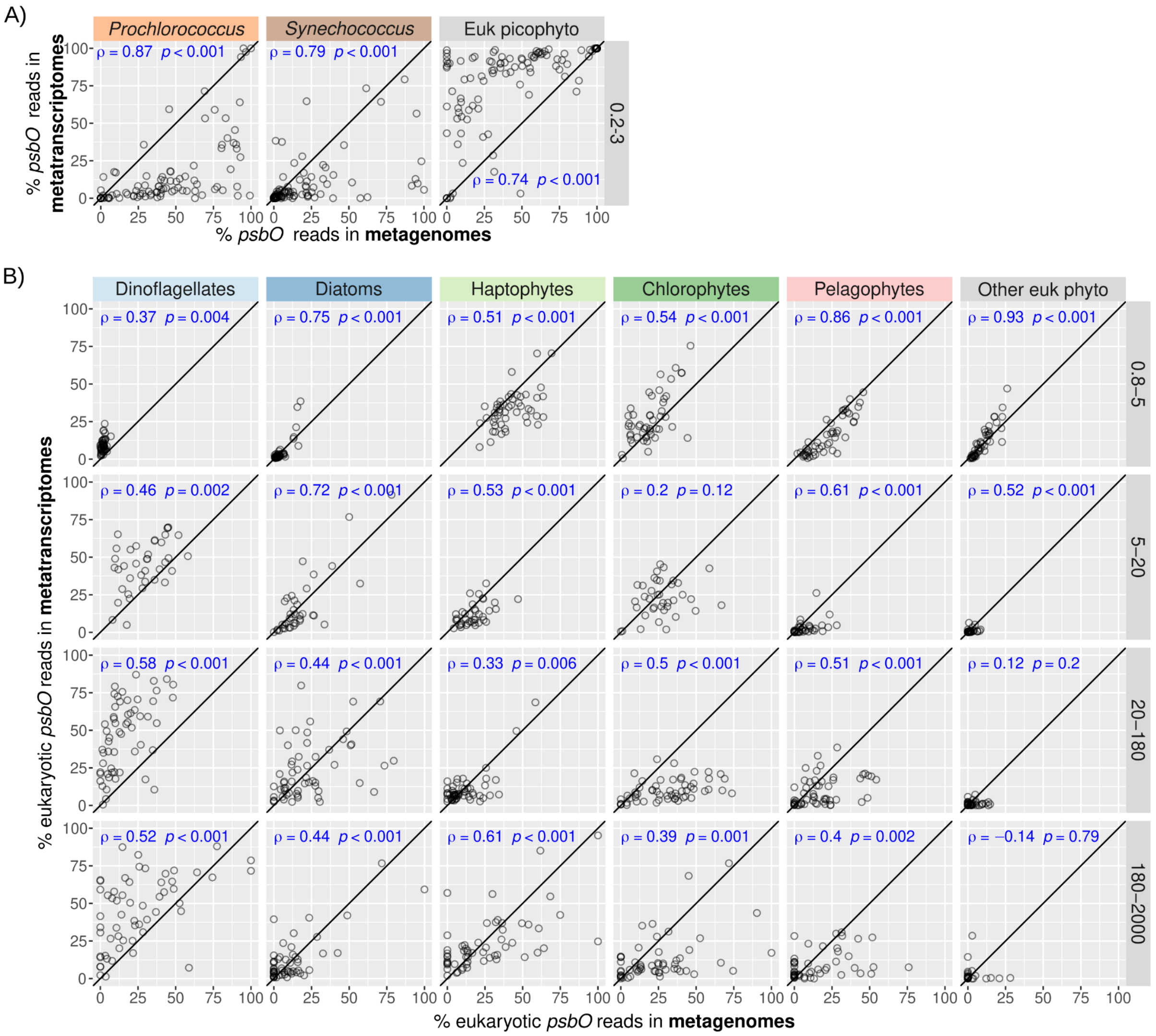

Figure S8: Comparison of relative abundances of *psbO* reads between metagenomes and metatranscriptomes of size fractionated samples. A) Picocyanobacteria and eukaryotic picophytoplankton (size fraction 0.2-3 µm). B) Eukaryotic phytoplankton in the large size fractions (0.8-5 µm, 5-20 µm, 20-180 µm, 180-2000 µm), with metagenomes compared to metatranscriptomes derived from poly-A RNA. Axis are in the same scale and the diagonal line corresponds to a 1:1 slope. Spearman's rho correlation coefficients and p-values are displayed in blue.

MOLECULAR ECOLOGY
RESOURCES

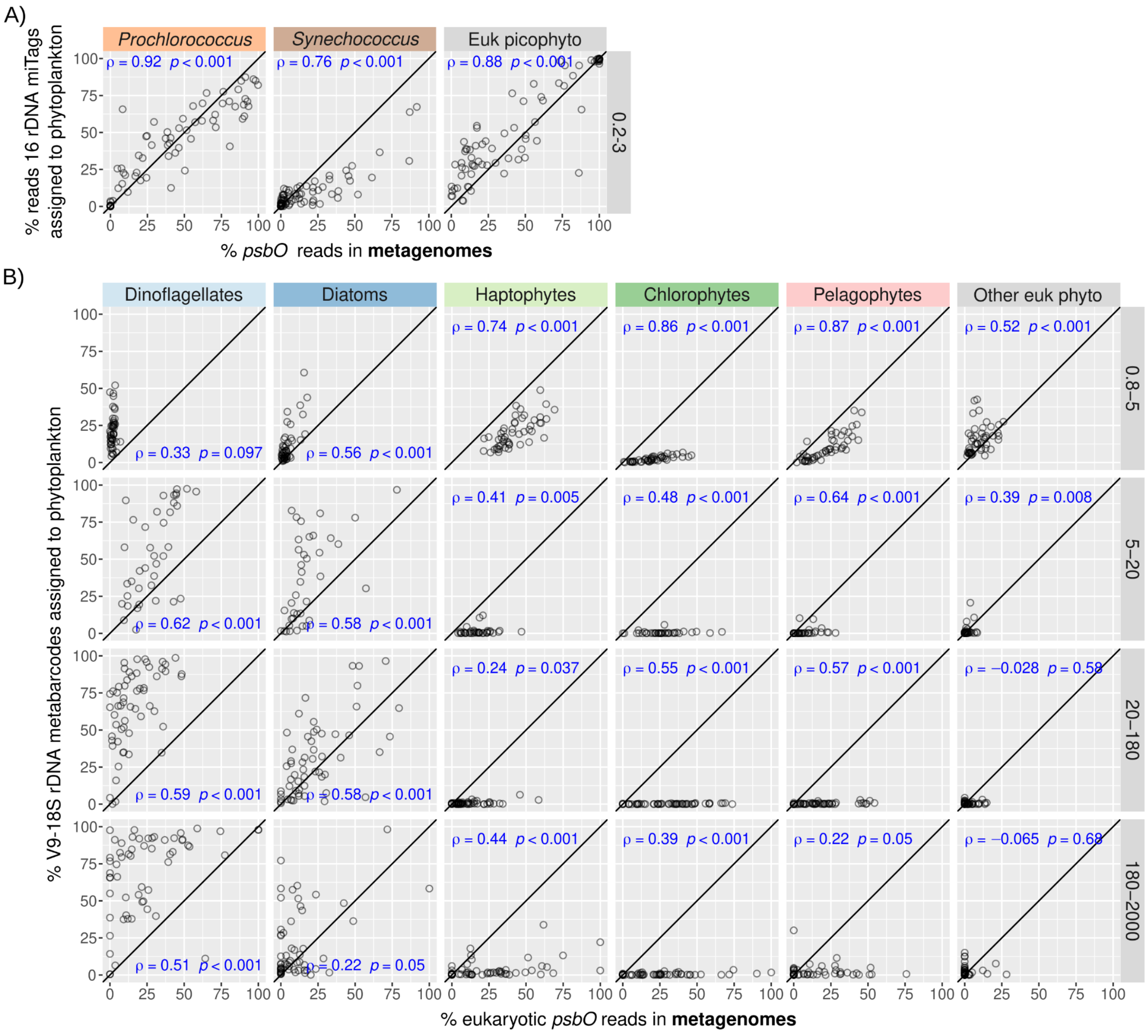

Figure S9: Comparison of relative read abundances of *psbO* and rRNA genes of size fractionated samples. A) Picocyanobacteria and eukaryotic picophytoplankton (0.2-3 µm) were analysed using the relative abundances for 16S rRNA gene miTags and for *psbO* metagenomic reads. B) Eukaryotic phytoplankton were analysed in the large size fractions (0.8-5 µm, 5-20 µm, 20-180 µm, 180-2000 µm) using the relative abundances for V9-18S rRNA gene amplicons and *psbO* metagenomic reads. Axis are in the same scale and the diagonal line corresponds to a 1:1 slope. Spearman's rho correlation coefficients and p-values are displayed in blue.

MOLECULAR ECOLOGY RESOURCES

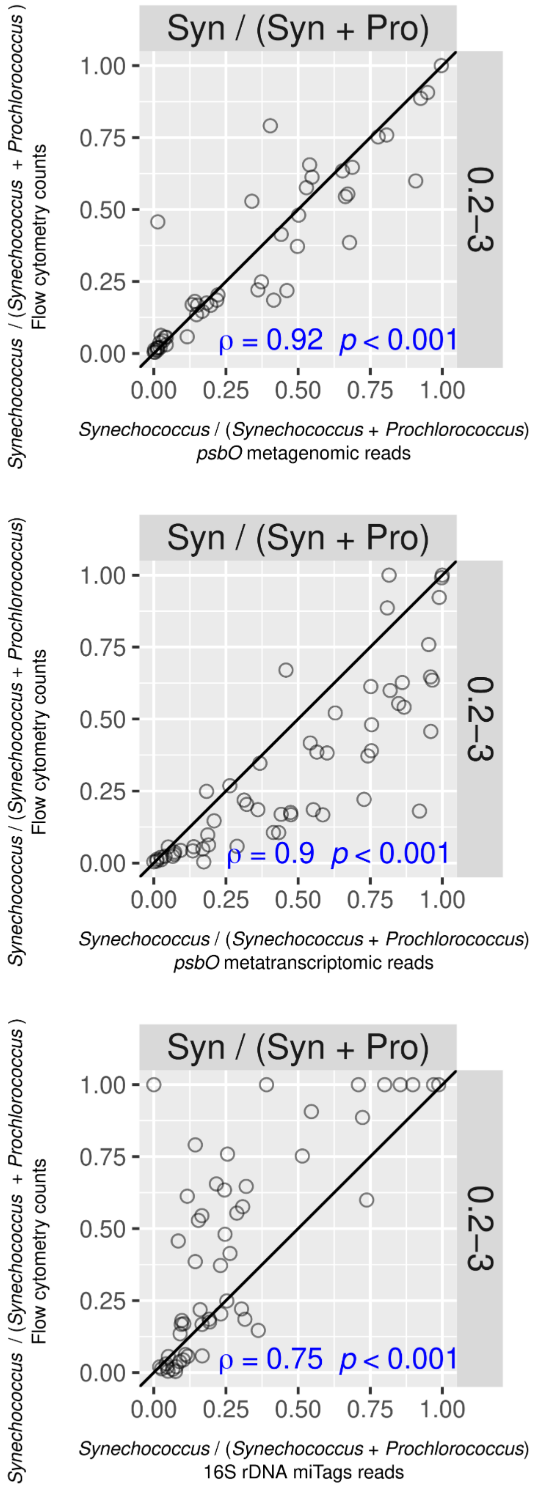

Figure S10: Correlation between the abundance ratio of the picocyanobacteria *Synechococcus* and *Prochlorococcus* obtained with different methodologies. The vertical axis corresponds to the ratio based on flow cytometry while the horizontal axis corresponds to the ratio based on *psbO* metagenomic reads (upper plot) or *psbO* metatranscriptomic reads (middle plot) or 16S miTAGs reads (bottom plot). Axis are in the same scale and the diagonal line corresponds to a 1:1 slope. Spearman's rho correlation coefficients and p-values are displayed in blue.

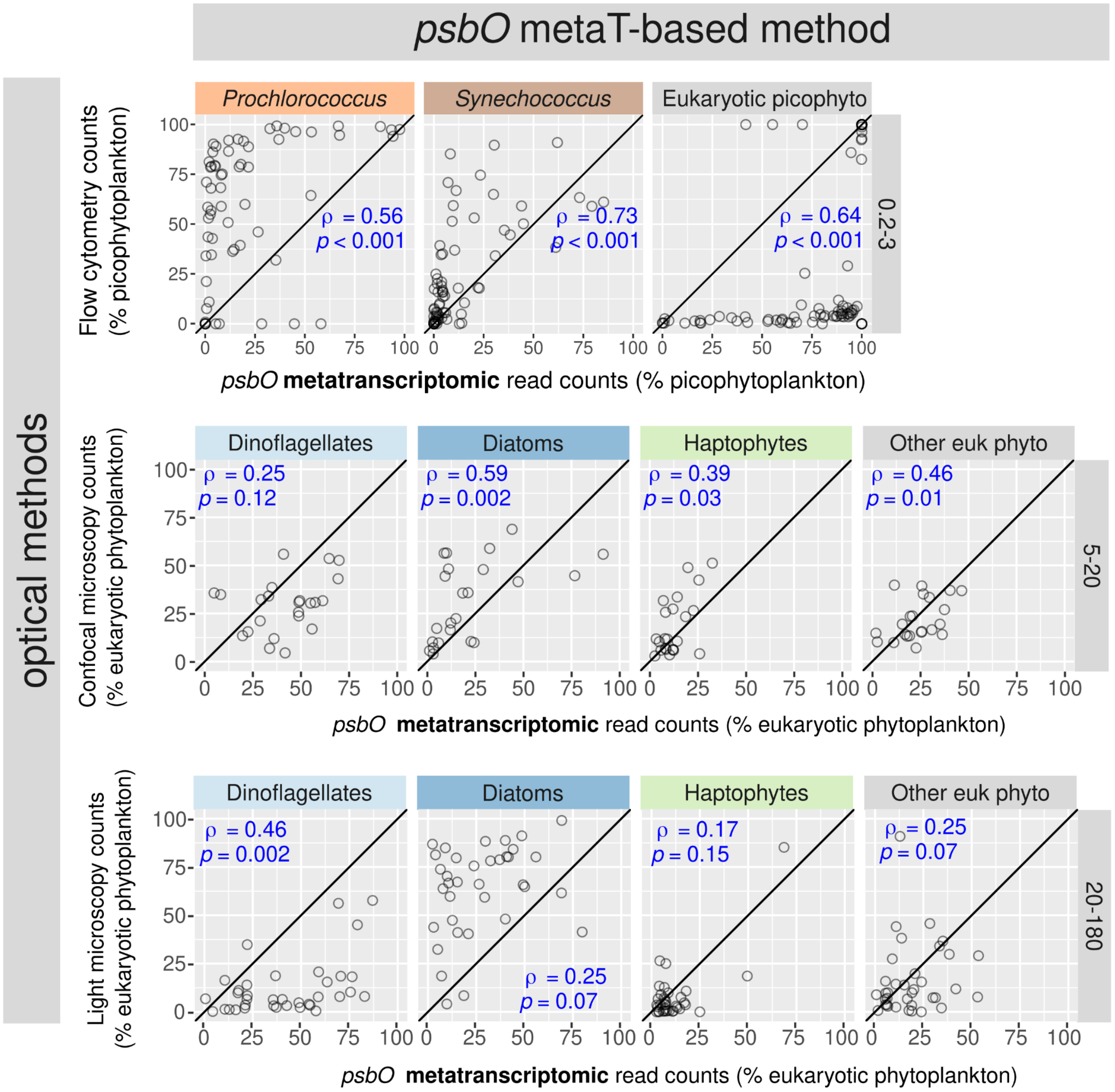

Figure S11: Correlation between relative abundances based on *psbO* metatranscriptomic reads against those based on optical quantifications of different phytoplankton groups. Upper panel: metatranscriptomic *psbO* relative abundances from picophytoplankton (size fraction 0.2-3 µm) were compared with flow cytometry counts (values displayed as % total abundance of picophytoplankton). Middle and lower panels: metatranscriptomic *psbO* relative abundances of eukaryotic phytoplankton were compared with confocal microscopy counts from size fraction 5-20 µm and light microscopy counts from size fraction 20-180 µm (values displayed as % total abundance of eukaryotic phytoplankton). Spearman correlation coefficients and p-values are displayed in blue. Axis are in the same scale and the diagonal line corresponds to a 1:1 slope.

MOLECULAR ECOLOGY
RESOURCES

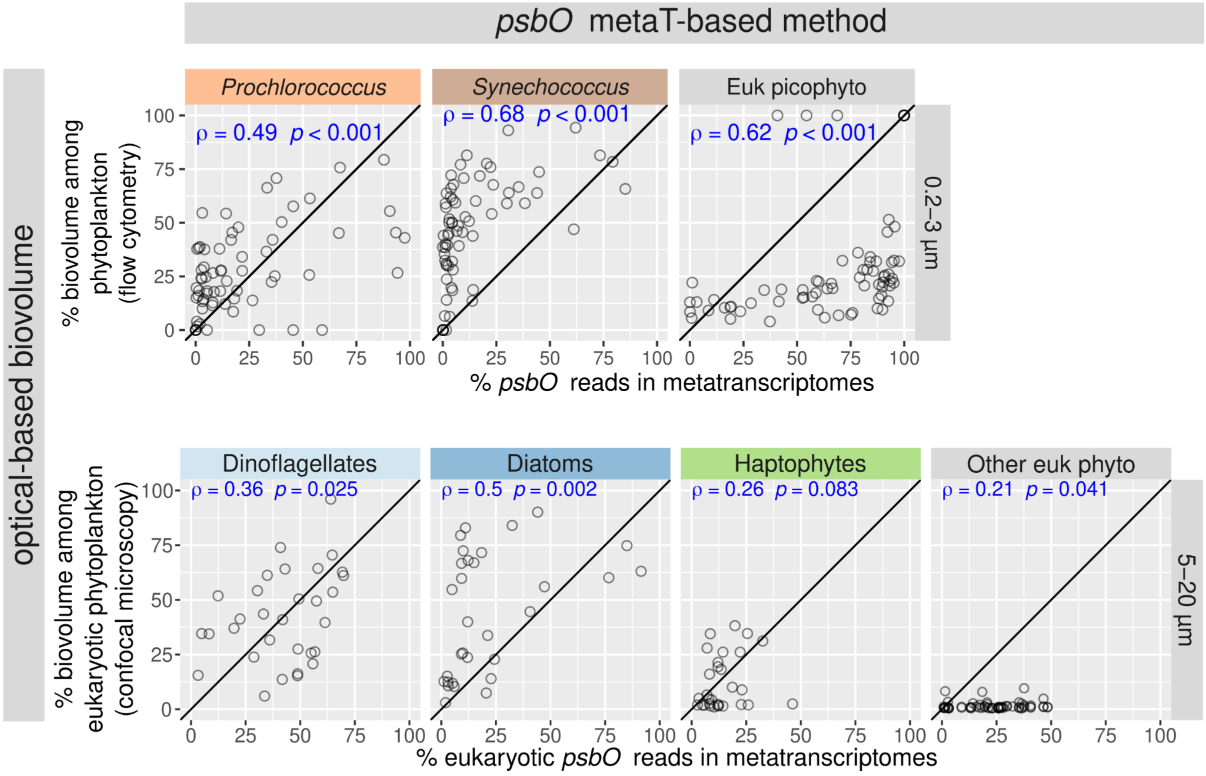

Figure S12: Correlation between relative biovolume (based on optical methods) and relative abundances based on *psbO* metatranscriptomic reads. The upper panel shows the correlations for picophytoplankton (size fraction 0.2-3 µm). The vertical axis corresponds to the relative biovolume based on flow cytometry (values displayed as % total biovolume of picophytoplankton), while the horizontal axis corresponds to relative read abundance based on *psbO* metatranscriptomic reads. The lower panel shows the correlations for nanophytoplankton (size fraction 5-20 µm). The vertical axis corresponds to the relative biovolume based on confocal microscopy quantification (values displayed as % total abundance of eukaryotic phytoplankton), while the horizontal axis corresponds to relative read abundance based on eukaryotic *psbO* metatranscriptomic reads. Spearman correlation coefficients and p-values are displayed in blue. Axis are in the same scale and the diagonal line corresponds to a 1:1 slope.

MOLECULAR ECOLOGY RESOURCES

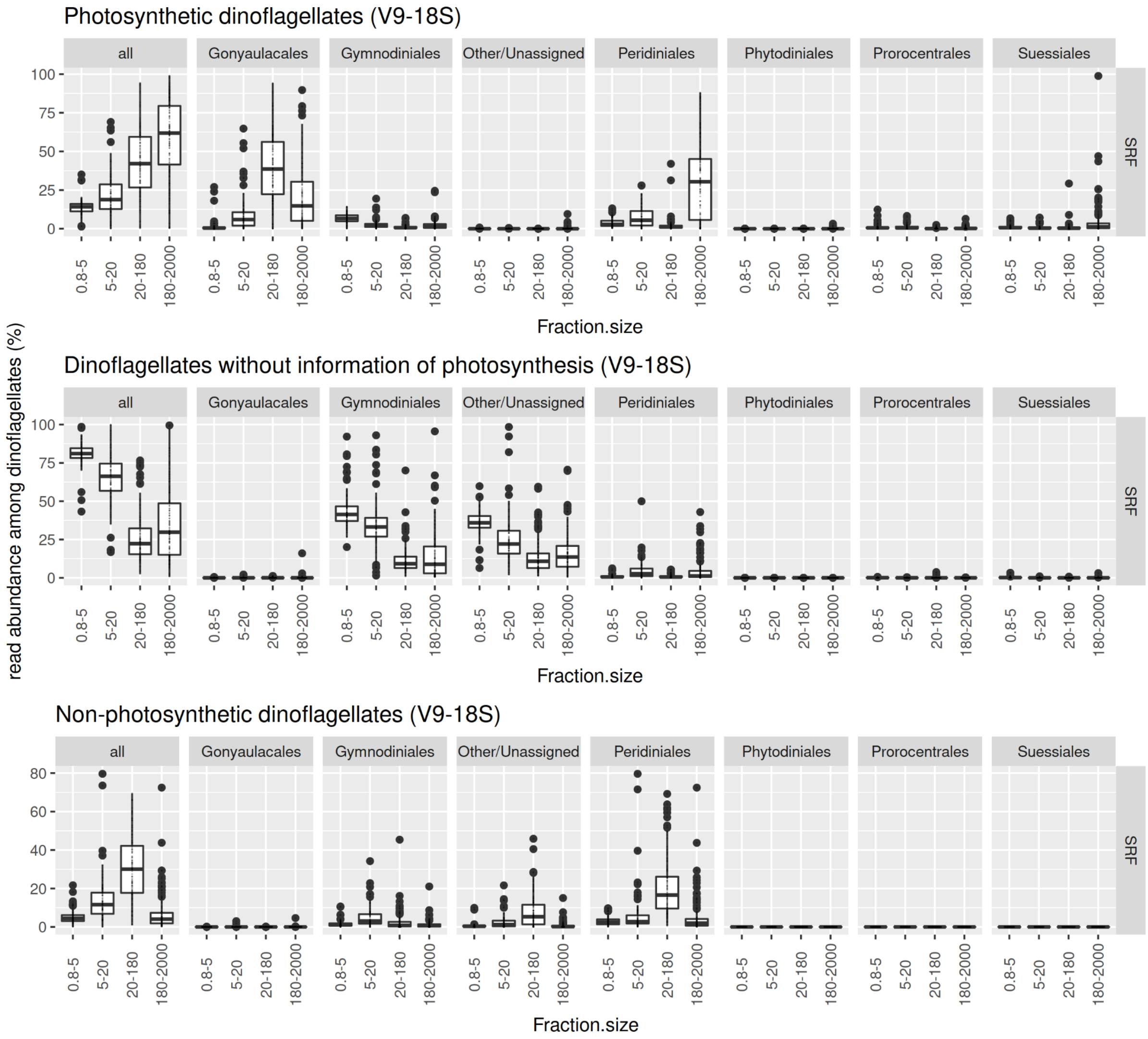

Figure S13: Variations in the abundance of phototrophs in the dinoflagellate community of different size fractions based on V9-18S rRNA gene metabarcoding. The plots show the relative abundance of dinoflagellates containing chloroplasts (upper panel) or not (lower panel) as well as those that cannot be classified (middle panel). Note that most of the reads that cannot be reliably classified as a chloroplast-containing taxon correspond to those reads mapping OTUs assigned either as “unknown dinoflagellate” or Gymnodiales order. A description of the trait classification can be found at http://taraoceans.sb-roscoff.fr/EukDiv/ and the trait reference database is available at https://zenodo.org/record/3768951#.YM4odnUzbuE.

MOLECULAR ECOLOGY RESOURCES

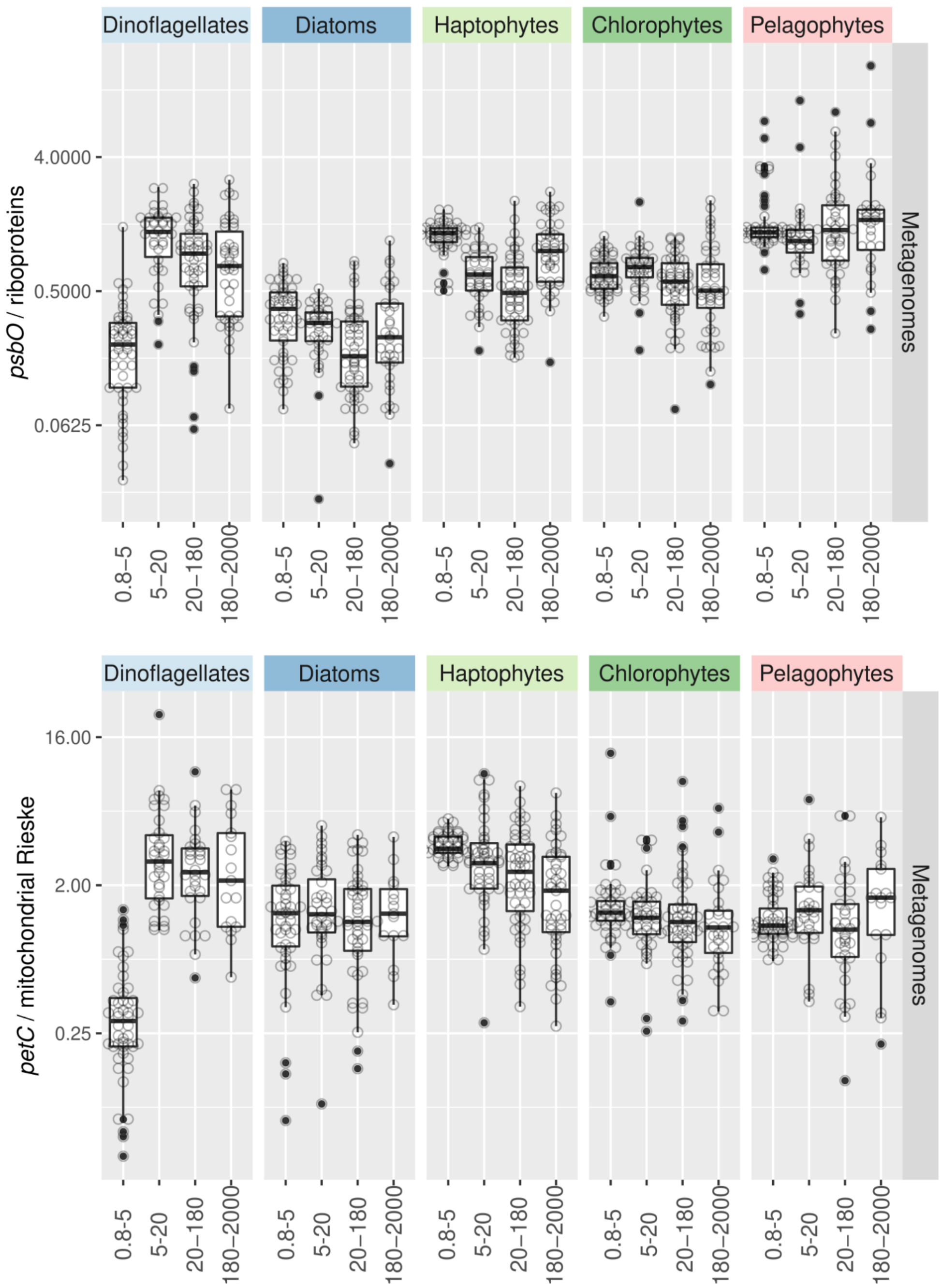

Figure S14: Variations in the abundance of phototrophs across size fractions based on different marker genes in metagenomes. The estimation were based on the ratio of metagenomic reads of photosynthetic *vs* housekeeping single-copy nuclear-encoded genes: *psbO* vs genes coding for ribosomal protein (upper panel), and the genes coding for the Rieske subunits of the Cyt *bc*-type complexes from chloroplasts and mitochondria (i.e., *petC* and its mitochondrial homologue) (lower panel).

MOLECULAR ECOLOGY
RESOURCES

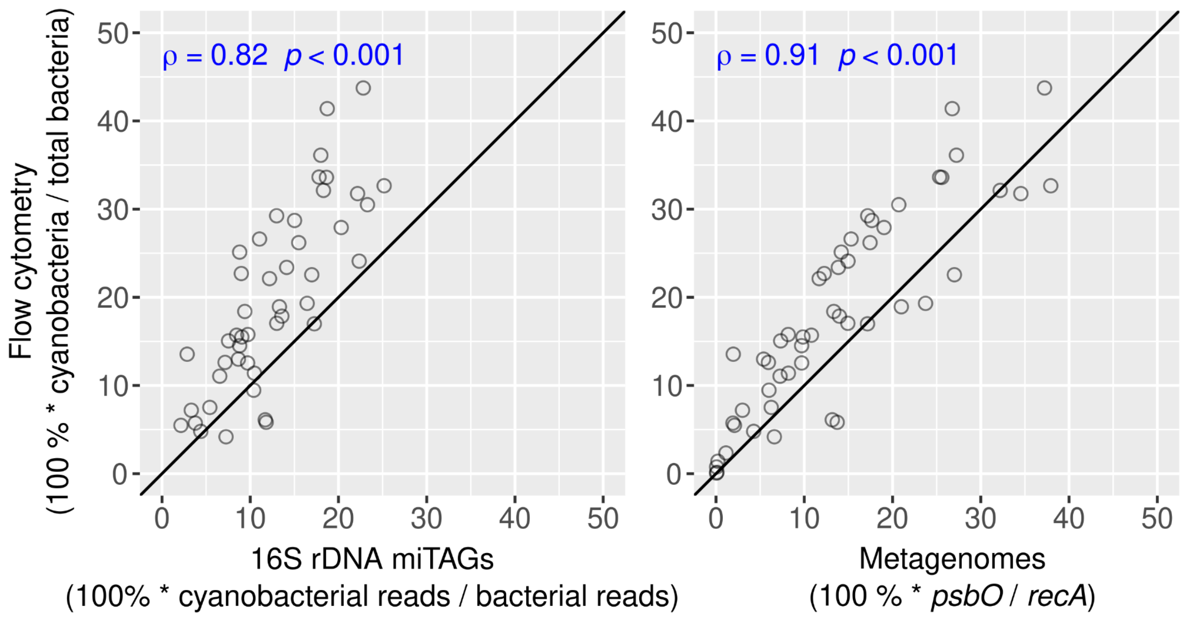

Figure S15: Congruence in the relative abundance of phototrophs among bacterioplankton in 0.2-3 size fraction based on different methods. The vertical axis corresponds to the relative cell abundance based on flow cytometry while the horizontal axis corresponds to the relative read abundance based on 16S miTags (left) or on the ratio of *psbO* to *recA* in metagenomes (right). Spearman correlation coefficients and p-values are displayed in blue. Axis are in the same scale and the diagonal line corresponds to a 1:1 slope.

MOLECULAR ECOLOGY
RESOURCES

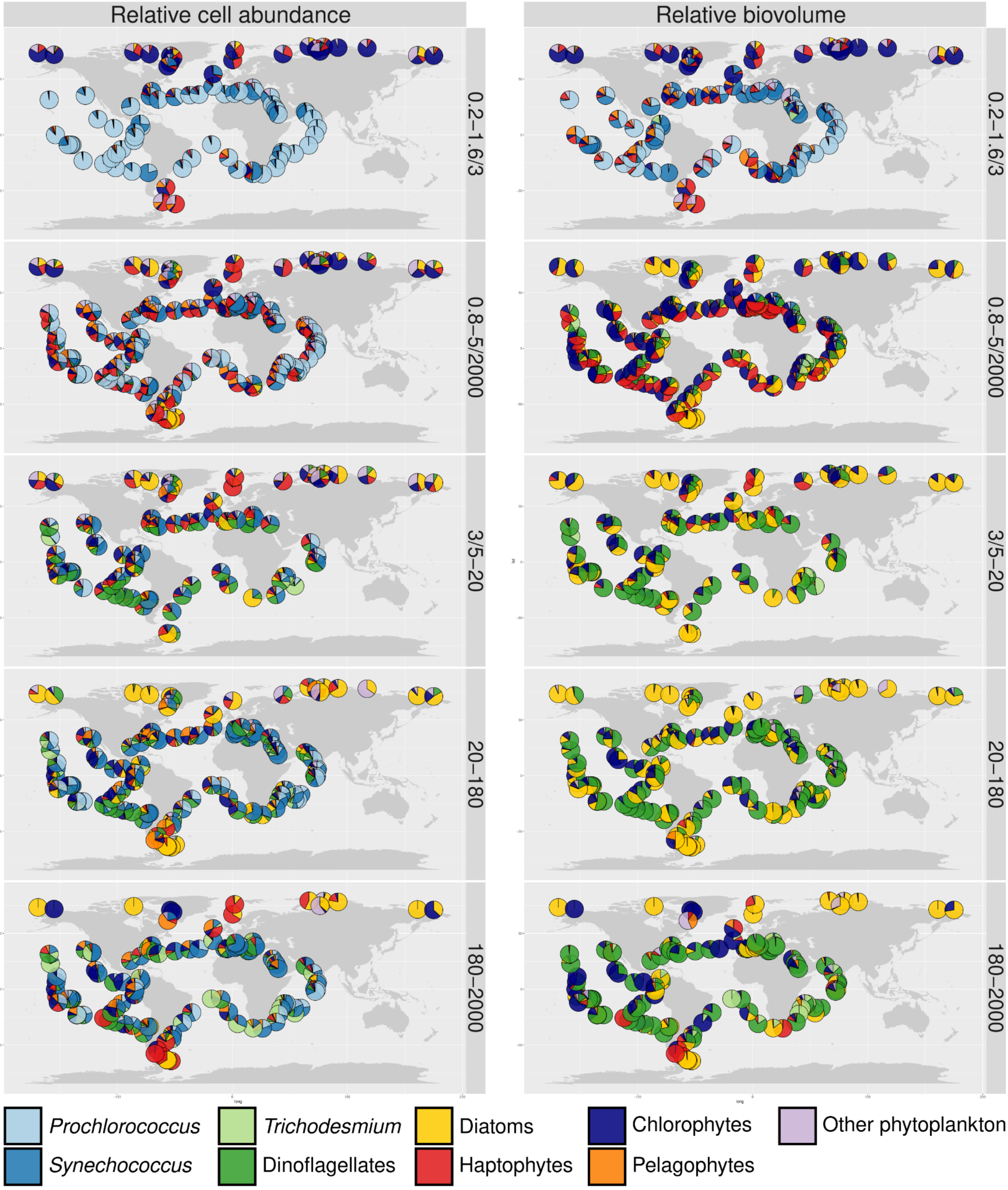

Figure S16: Global biogeographical patterns of relative cell abundance and biovolume for marine phytoplankton in surface waters. A) Relative cell abundances of the main cyanobacteria and eukaryotic phytoplankton based on *psbO* counts in metagenomes derived from different size-fractionated samples. B) Relative biovolume of the main cyanobacteria and eukaryotic phytoplankton based on *psbO* counts correctected by the mean cell biovolume for each taxon (based on optical measurements in *Tara* Oceans samples).

MOLECULAR ECOLOGY
RESOURCES

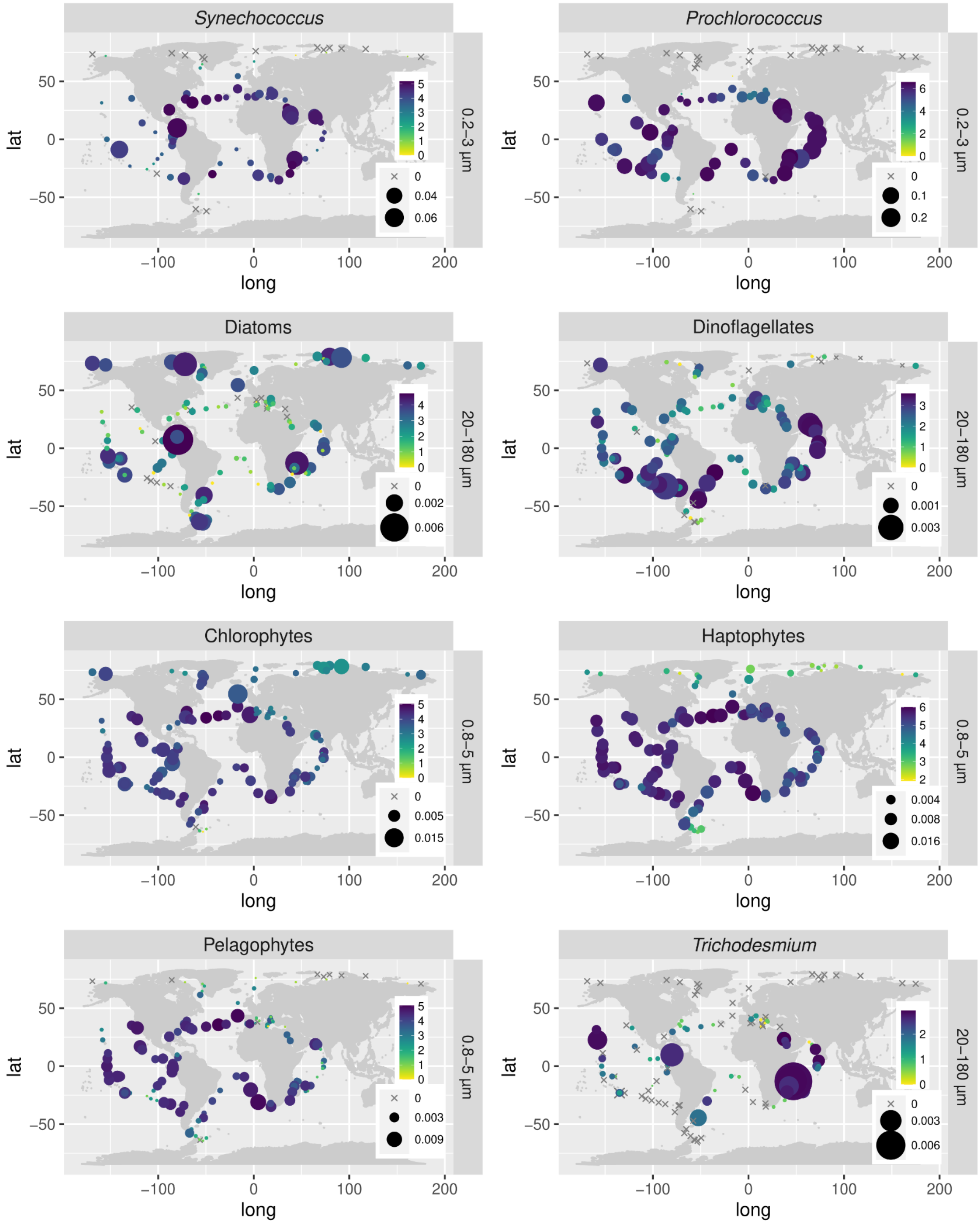

Figure S17: Global biogeographical patterns for main groups of marine phytoplankton in surface waters. The bubbles sizes vary according to the *psbO* relative abundance of the main cyanobacteria and eukaryotic phytoplankton in metagenomes, while color code corresponds to the Shannon index values. Relative abundance values are displayed as rpkm (reads per kilobase per million mapped reads). Only the size fraction where the corresponding taxon was prevalent is shown: 0.2-3 µm for picocyanobacteria, 20-180 µm for diatoms and dinoflagellates, and 0.8-5 µm for chlorophytes, haptophytes and pelagophytes. The corresponding analysis for the whole phytoplankton community in each size fraction is displayed in Figure 7.
